# Inadvertent Paralog Inclusion Drives Artifactual Topologies and Timetree Estimates in Phylogenomics

**DOI:** 10.1093/molbev/msz067

**Published:** 2019-03-23

**Authors:** Karen Siu-Ting, María Torres-Sánchez, Diego San Mauro, David Wilcockson, Mark Wilkinson, Davide Pisani, Mary J O’Connell, Christopher J Creevey

**Affiliations:** 1 Institute for Global Food Security, School of Biological Sciences, Queen’s University Belfast, Belfast, United Kingdom; 2 School of Biotechnology, Dublin City University, Glasnevin, Dublin, Ireland; 3 Dpto. de Herpetología, Museo de Historia Natural, Universidad Nacional Mayor de San Marcos, Lima, Perú; 4 Department of Biodiversity, Ecology, and Evolution, Complutense University of Madrid, Madrid, Spain; 5 Institute of Biological, Environmental and Rural Sciences, Aberystwyth University, Aberystwyth, United Kingdom; 6 Department of Life Sciences, Natural History Museum, London, United Kingdom; 7 Life Sciences Building, University of Bristol, Bristol, United Kingdom; 8 School of Biology, Faculty of Biological Sciences, University of Leeds, Leeds, United Kingdom; 9 School of Life Sciences, University of Nottingham, University Park, United Kingdom; 10 Department of Neuroscience, Spinal Cord and Brain Injury Research Center and Ambystoma Genetic Stock Center, University of Kentucky, Lexington, KY

**Keywords:** phylogenomics, orthology, paralogy, Lissamphibia, timetree

## Abstract

Increasingly, large phylogenomic data sets include transcriptomic data from nonmodel organisms. This not only has allowed controversial and unexplored evolutionary relationships in the tree of life to be addressed but also increases the risk of inadvertent inclusion of paralogs in the analysis. Although this may be expected to result in decreased phylogenetic support, it is not clear if it could also drive highly supported artifactual relationships. Many groups, including the hyperdiverse Lissamphibia, are especially susceptible to these issues due to ancient gene duplication events and small numbers of sequenced genomes and because transcriptomes are increasingly applied to resolve historically conflicting taxonomic hypotheses. We tested the potential impact of paralog inclusion on the topologies and timetree estimates of the Lissamphibia using published and de novo sequencing data including 18 amphibian species, from which 2,656 single-copy gene families were identified. A novel paralog filtering approach resulted in four differently curated data sets, which were used for phylogenetic reconstructions using Bayesian inference, maximum likelihood, and quartet-based supertrees. We found that paralogs drive strongly supported conflicting hypotheses within the Lissamphibia (Batrachia and Procera) and older divergence time estimates even within groups where no variation in topology was observed. All investigated methods, except Bayesian inference with the CAT-GTR model, were found to be sensitive to paralogs, but with filtering convergence to the same answer (Batrachia) was observed. This is the first large-scale study to address the impact of orthology selection using transcriptomic data and emphasizes the importance of quality over quantity particularly for understanding relationships of poorly sampled taxa.

## Introduction

It has often been argued that including ever larger numbers of genes contributes to more robust phylogenetic analyses (Graybeal 1998; Kim 1998; Rokas and Carroll 2005; Philippe et al. 2011; San Mauro et al. 2012; Chen et al. 2015; Irisarri et al. 2017) motivating the use of phylogenomic-scale data to address long-standing controversies across all lineages of the tree of life (Delsuc et al. 2005; Thomson and Shaffer 2010; Giribet 2016). This has been driven in practice primarily by access to cheaper and higher-throughput sequencing technologies generating both genomic and, increasingly, transcriptomic data for previously unsampled organisms. Although the technologies and types of data have moved forward, little has changed in the practical approach to ortholog detection since the days when phylogenetic reconstruction was dominated by Sanger sequencing (Koonin 2005; Gabaldón 2008). This has consequences for several stages of the phylogenomic analytical pipeline because many assumptions that could previously have been made about the sequence data may no longer hold (da Fonseca et al. 2016). For instance, transcriptomic data represent a snapshot in time of the genes expressed by an organism in the tissue sampled, this means that highly expressed genes may have more reads allowing better reconstruction of their transcripts compared with more lowly expressed genes. Furthermore, the absence of a gene from a transcriptomic data set may either mean it was not expressed at the time or tissue of sampling, or that it is not found in the organism. Compounding this, in many eukaryotic species, temporal or tissue-specific alternative splicing of exons may result in alternative isoforms of genes being transcribed into mRNA, resulting in single-copy genes giving the appearance of being multicopy or vice versa. Finally, even with ideal genomic data, it can be extremely challenging to recognize genes that have been in single copy since the Last Common Ancestor (LCA) of the species under study from those genes that were in multiple copies in the LCA but are in single copy in extant organisms due to subsequent lineage-specific loss (i.e., paralogs retained in single copy).

These and other issues can result in paralogs (whose evolutionary history reflects a mix of speciation and gene duplication events) being falsely identified as orthologs or vice versa in phylogenomic studies, introducing confounding phylogenetic signals from genes with different histories. However, not every phylogenetic question requires the analysis of genome-scale data sets to be resolved, indeed simulations (Zwickl and Hillis 2002; Salamin et al. 2005) and empirical studies (Erdős et al. 1997) indicate that moderate sized data sets can be sufficient to reconstruct trees even with large numbers of taxa (Steel 2007), suggesting that approaches that prioritize quality over quantity should be considered. Although it is likely that inadvertent inclusion of paralogs in genome-scale studies could result in decreased support for the correct relationship, it is not clear if they could cause highly supported alternative relationships to be retrieved instead, even when they form a minority of all the genes used in the analysis. If true, this may explain recent findings where, regardless of the application of increasingly large genomic data sets, conflicting hypotheses are still retrieved (Philippe et al. 2011; Chen et al. 2015; Shen et al. 2017). Although several studies have investigated the effects of missing data (Roure et al. 2013; Jiang et al. 2014; Streicher et al. 2016) and inadequate models (Morgan et al. 2013; Pisani et al. 2015; Feuda et al. 2017), the extent to which poor ortholog selection impacts final topologies and timetree estimations in phylogenomic studies is currently not known.

**Fig. 1.**
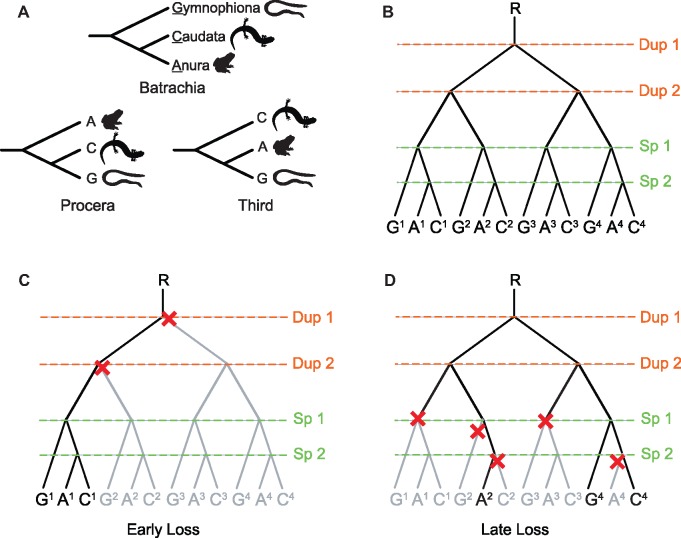
Evolutionary relationships in the Lissamphibia and hypotheses of ancient duplications, speciation, and loss of gene copies. (*A*) The three possible hypothetical phylogenetic relationships (Batrachia, Procera, and a Third topology) among the living lineages of Lissamphibia: Gymnophiona (G), Anura (A), and Caudata (C). (*B*) Hypothesis of two rounds of ancient duplication events (“DUP” in orange) prior to the Lissamphibia speciation events (“SP” in light green) that give rise to multiple gene copies assuming the most accepted hypothesis in the literature, Batrachia. In superscript are the IDs of the different copies of the gene that have arisen following the duplication events. (*C*) Framework of “Early Loss” resulting from gene deletions (red crosses) prior to the speciation events. In this example, all gene copies are orthologs and retrieve Batrachia. (*D*) Framework of “Late Loss” resulting from gene deletions (red crosses) after the speciation events. In this example, there is a mix of ortholog and paralog genes which retrieve Procera. For figures (*B*–*D*), lineages in black represent retained copies and lineages in gray represent lost copies.

It is generally accepted that gene duplication events are an important component of vertebrate evolution (Sidow 1996; Donoghue and Purnell 2005; Nakatani et al. 2007; Cañestro et al. 2013). A large body of work provides evidence that genomes of ancient vertebrates underwent two rounds of whole genome duplications early in their evolution (i.e., the 2R hypothesis—fig. 1*B* ) (Ohno 1970; Holland et al. 1994; Meyer and Schartl 1999; Nakatani et al. 2007). It is likely that over time many of the resulting paralogs became pseudogenes and were lost (Meyer and Schartl 1999). The rate of loss would have been related to the selective pressure (or lack thereof) against redundant copies of the genes. Using this well-established phenomenon, we can make some inferences about the scenarios that can result in single-copy genes in extant species. For genes where duplicate copies were disadvantageous (for instance because of gene dosage effects [Cañestro et al. 2013; Gout and Lynch 2015]), loss is likely to have occurred early—defined here as “early loss” events. It is possible that early loss events occurred prior to any subsequent speciation events and they are most likely to represent “true deep orthologs” in extant species (fig. 1*C*). However, for genes where duplicate copies were neither positively nor negatively selected, loss of duplicates could have occurred much later—defined here as “late loss” events. These late loss events can occur independently in multiple subsequent lineages and are most likely to result in misclassified paralogs in extant species (fig. 1*D*). Overall, single-copy genes in extant jawed vertebrates likely consist of a mixture of both orthologs and paralogs, a product of both early and late-gene loss events following ancient duplication events. The application of single-copy genes derived from a mixture of early and late loss events to phylogenomic studies could be fueling strongly supported conflicting topologies within the jawed vertebrate group.

The Lissamphibia, a group of jawed vertebrates that comprise the three orders of extant Amphibia: Anura (frogs and toads), Caudata (salamanders and newts) and Gymnophiona (caecilians), is a case in point. For many years, the evolutionary relationships among these three orders have remained a controversial question in vertebrate evolution (Feller and Hedges 1998; Zardoya and Meyer 2001; Ruta and Coates 2007; Chen et al. 2015; Shen et al. 2017) and citations therein) with two main conflicting hypotheses. The Procera hypothesis proposes a sister-group relationship between Gymnophiona and Caudata, to the exclusion of Anura (fig. 1*A*) (Feller and Hedges 1998; Vallin and Laurin 2004), whereas the Batrachia hypothesis posits a sister-group relationship between Caudata and Anura, to the exclusion of Gymnophiona (fig. 1*A*) (Milner 1988; Zardoya and Meyer 2001; Ruta and Coates 2007). In theory, a third topology (proposing a sister group between the Anura and Gymnophiona to the exclusion of Caudata) is phylogenetically possible, but it has never been supported by morphological or concatenated molecular data sets (fig. 1*A*). Such conflicts in the Lissamphibia have been attributed to uninformative data and poor taxon sampling (Cannatella et al. 2009), but if whole genome duplications occurred prior to the LCA of Amphibia, then the “worst case” scenario for phylogenomic analyses would be that for single-copy genes in extant species, multiple copies were present in the LCA of Lissamphibia and the extra copies were lost following the separation of Anura, Gymnophiona, and Caudata (i.e., Late Loss) (fig. 1*D*). This would result in support for all three possible topologies in the set of genes that have undergone a “late loss,” however the relative influence of such ortholog misspecification on the resulting topology and time estimates is currently unknown.

In order to address this, we have gathered published genomic and transcriptomic data for carefully selected taxa and generated de novo transcriptomic data for targeted species where data were lacking. Taking a phylogenomic approach and focusing on the Lissamphibia group, we test the hypothesis that inadvertent paralog inclusion is driving the observed highly supported yet conflicting topologies and we examine their impact on divergence time estimates.

## Results

### Data Sequenced and Processed

The genomic and transcriptomic data sets retrieved and de novo sequenced for the 33 species included in the study are outlined with its corresponding assembly statistics in supplementary table S1, Supplementary Material online, and are available in NCBI under the Bioproject IDs PRJNA387587 and PRJNA430346. Overall, the transcriptomes assembled for the six species of Anura de novo sequenced for this study ranged from 85,877 to 368,483 contigs per taxon, with average N50 of 769. The previously published transcriptomic data for the remaining 12 taxa downloaded from various sources ranged from 56,401 to 451,790 contigs per taxon and average N50 of 1,310. Information for all assemblies included in this study are summarized in supplementary table S1, Supplementary Material online. Coding DNA sequences (CDS) predicted for the assembled transcriptomes after filtering for redundancies ranged between 19,811 and 121,567 CDS per taxon, with an average of 51,268 CDS per taxon. Downloaded CDS from genomic data averaged about 19,931 per taxon, ranging between 10,402 and 31,066 after filtering. Following sequence alignment, tree estimation, filtering for duplicates, subsequent separation from multigene families, and tests for sequence saturation (see Materials and Methods), we obtained a set of 2,656 single-copy gene families for the phylogenomic analyses. These were then filtered to identify possible hidden paralogy based on the premise that some of the single-copy gene families may be paralogous due to ancient duplication events that occurred prior to the LCA of both the ingroups and outgroups (fig. 1*B*). In this scenario, subsequent lineage-specific loss of extra copies of the genes occurring after LCA of the taxa in the study (late loss) could lead to single-copy gene families which violate the assumption of deep orthology across the jawed vertebrates (including the outgroups) (fig. 1*D*). In contrast, those genes that are single-copy due to loss of extra copies prior to the LCA of the taxa in the study (early loss) are more likely to represent deep orthologs in the jawed vertebrates (fig. 1*C*). If paralogy is driving the observed alternative relationships in the base of the Lissamphibia, identifying and removing genes where “incontestable” clans in the outgroups are violated should result in an enrichment in orthologs in the data set overall and an increase in the phylogenetic signal for the true topology (see Materials and Methods for more details). We implemented this approach in a tool called “Clan_Check” (https://github.com/ChrisCreevey/clan_check; last accessed April 1, 2019).

Following on from this framework, we generated four test data sets (named after the number of families that it contains, see Materials and Methods for more details): 1) all 2,656 gene families (Data set 2656); 2) gene families enriched for orthologs after applying Clan_Check (Data set 2019); 3) those gene families from Data set 2019 that had at least one representative from the Anura, Caudata, Gymnophiona, and the outgroups (i.e., those genes that have the potential to address the Lissamphibia question) (Data set 348); those gene families from Data set 2656 under the same constraint as (3), that is, potentially informative for the Lissamphibia question but without enriching for orthologs (Data set 768). The distribution of gene-family sizes for each of the four data sets is shown in supplementary figure S1, Supplementary Material online.

### The Amphibia Are Monophyletic, but Procera Is an Artifact of Inadvertent Paralog Inclusion

**Fig. 2.**
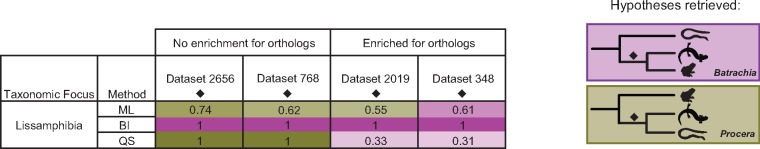
Resulting topologies for the extant lineages of Lissamphibia with all four data sets tested. Data sets 2656 and 768 have no enrichment of orthologs (no Clan_Check filter) and Data sets 2019 and 348 have enrichment of orthologs (after Clan_Check filter). Alternative hypotheses retrieved (shown on the right side) for Lissamphibia are represented by a different color in the table: Batrachia is in purple, Procera is in olive green. Numbers in the table are the support values for each data set and method used, and these are represented by color intensity (higher support for the node, more intense color). The complete set of results for the entire 33 taxa can be found in supplementary figures S2–S13, Supplementary Material online. Abbreviations: “ML” stands for maximum likelihood, “BI” for Bayesian inference, and “QS” for quartet-based supertree method. In the case of ML, support values correspond to bootstrap proportions, for BI they represent Posterior Probability (scaled over 1), and for QS they represent local posterior PP (scaled over 1).

As expected and in agreement with most previous work, the monophyly of Amphibia was confirmed with maximal support in analyses of all four data sets irrespective of the phylogenetic method used (fig. 2 and supplementary figs. S2–S13, Supplementary Material online). However, there was conflict in the inferred relationships within the Lissamphibia (fig. 2 and supplementary figs. S2–S13, Supplementary Material online). Our phylogenomic analyses of Data set 2656 (i.e., the complete set of gene families after filtering for saturation) using maximum likelihood (ML), and quartet-based supertrees (QS) all supported the Procera hypothesis (Caudata + Gymnophiona) with high support: 74% bootstrap support (BS) with the ML approach and 1.0 local posterior probability (LPP) with the QS method (fig. 2 and supplementary figs. S2–S4, Supplementary Material online). Only the Bayesian inference (BI) using the CAT-GTR model in Phylobayes supported the Batrachia hypothesis, and with maximal posterior probability (PP = 1.0). These results are summarized in figure 2.

However, using Data set 2019 (generated following filtering potential paralogous gene families from Data set 2656 with Clan_Check as described in Materials and Methods), QS favors the Batrachia hypothesis, albeit with low support (0.33 LPP) as does BI with maximal support (PP = 1.0) (fig. 2 and supplementary figs. S5–S7, Supplementary Material online). Using Data set 348 (selecting only those genes from Data set 2019 that have the potential to address the Lissamphibia question, see Materials and Methods), all methods retrieved the Batrachia hypothesis with variable support (ML: 65% BS; BI: 1.0 PP; QS: 0.33 LPP) (fig. 2 and supplementary figs. S8–S10, Supplementary Material online). This confirms our hypothesis that filtering for potential “late loss” genes would result in enrichment for genes supporting one of the topologies and suggests that inadvertent inclusion of paralogs may explain the support for the Procera hypothesis obtained in some previous phylogenomic studies (Fong et al. 2012; Chen et al. 2015).

### Procera Is Not an Artifact of Missing Data

To test whether the observed convergence toward the Batrachia hypothesis was linked to the reduction of missing data, we applied the same phylogenetic methods to Data set 768, which contains genes families that address the Lissamphibia question but with no filtering for hidden paralogs (equivalent to Data set 348 except without filtering). Data set 768 has only 36% missing data (compared with Data sets 2656, 2019, and 348 with 68%, 76%, and 43% missing data, respectively), hence it provides the opportunity to identify if removal of paralogous gene families rather than missing data caused the differences in the results observed between Data set 2656 and both Data set 2019 and Data set 348. As with all other data sets, the monophyly of Lissamphibia was recovered with maximal support (fig. 2 and supplementary figs. S11–S13, Supplementary Material online). However, for phylogenetic relationships within Lissamphibia, the analysis supported the Procera hypothesis in two methods (ML with 62% BS and QS with 1.0 LPP), while once again BI retrieved the Batrachia hypothesis with 1.0 PP (fig. 2 and supplementary figs. S11–S13, Supplementary Material online). These results demonstrate that missing data is not responsible for the support for the Procera hypothesis in the ML and QS approaches.

### The Proportion of Genes Supporting Batrachia Increases Following Filtering for Putative “Late Loss” Genes

An example of a “worst case” scenario for phylogenomic analyses is where all single-copy genes in extant species are a result of late-gene loss following two rounds of ancient duplications (fig. 1*D*). It is straightforward to extrapolate for this scenario all 64 possible combinations of surviving gene copies in subsequently single-copy gene families and determine the topologies that they would support. This random late loss scenario would result in 38% (24) of genes supporting the true species topology and 31% (20) supporting each of the other two possible topologies. Worryingly, in this scenario, only 4 of the combinations supporting the true topology would represent real orthologs, the remaining 20 genes are paralogs that support the true topology simply by chance. This worst case scenario is unlikely however as support for the true species topology is expected to be higher, and proportional to how many genes are single-copy due to early loss (and therefore most likely true single gene orthologs).

In order to investigate if support for the all three topologies for the base of Lissamphibia exists in the underlying data, for each of the 768 single-copy genes capable of addressing the question of the relationship between the three living orders of Amphibians, we carried out Approximately Unbiased (AU) tests (Shimodaira 2002) of the three possible topologies (Batrachia, Procera, and the Third unnamed topology) (fig. 1*A*). For the 90 genes with enough phylogenetic signal to significantly reject all but one topology 45% (41) support Batrachia, 36% (33) support Procera and 17% (16) support the third topology (table 1). When examining the subset enriched for orthologs following Clan_Check filtering (Data set 348), for the 35 that significantly rejected all but one topology, support for Bactrachia increased to 51% (18), whereas only 28% (10) support Procera and 20% (7) support the third topology. Interestingly, examination of the remaining 420 genes that were identified as putative late loss paralogs by Clan_Check (see supplementary table S2, Supplementary Material online, for a detailed overview of the specific incontestable clans violated by each of these gene families) revealed that, of the 55 that significantly rejected all but one topology, 42% (23) support Batrachia, 42% (23) support Procera, and 16% (9) support the third topology. These results are consistent with our hypothesis that paralogy is a problem at this branch and that filtering for violations of incontestable relationships in outgroups enrich for genes supporting the species phylogeny (Batrachia).

**Table 1.** Results from AU Tests of All Three Possible Topologies with the 768 Gene Families Capable of Addressing the Root of the Lissamphibia.

Gene-Family Data Set	Number with Single Accepted Tree^a^	Hypothesis Favored		
		Percentage Supporting Batrachia (number)	Percentage Supporting Procera (number)	Percentage Supporting Third Topology (number)
All 768 gene families	90	45% (41)	36% (33)	17% (16)
348 genes passing Clan_Check	35	51% (18)	28% (10)	20% (7)
420 gene failing Clan_Check	55	42% (23)	42% (23)	16% (9)

a Number of gene families that had enough phylogenetic information to reject all hypotheses except one.

### Clan_Check Filters for Paralogy without Being Biased by Incomplete Lineage Sorting, Genes with Long Branch Attraction, Compositional Heterogeneity or Specific Gene Ontology

To assess the ability of Clan_Check to filter for paralogs, we used Simphy v1.0 (Mallo et al. 2016) to simulate 100 replicates of two data sets based on taxon frequencies and gene-family sizes of Data set 768. The first simulated data set was generated under a model of only incomplete lineage sorting (ILS), whereas the second simulated data set was generated under a model of early duplications followed by ILS and late loss (generating paralogous single-copy gene families). All the resulting trees from the simulations are available at the Open Science Framework (https://osf.io/hv5bk/, last accessed March 12, 2019). Under ILS only, Clan_Check identified that on average only 5% of incontestable clans were violated, whereas under ILS with early duplications and late loss Clan_Check identified that on average 72% of incontestable clans were violated (supplementary fig. S14, Supplementary Material online). We also tested with the real data whether the Clan_Check filter could be biased toward branch length, compositional heterogeneity, or gene function using Data set 768. We found that there was no significant difference (Wilcoxon test, *P* value = 0.1616) in the distribution of average branch lengths in those gene families identified as putative orthologs or paralogs by Clan_Check (supplementary fig. S15, Supplementary Material online), suggesting that long branch attraction was not a systematic bias in the resulting gene sets. Similarly, there was no significant difference (Wilcoxon test, *P* value = 0.4887) in the number of gene families with compositional heterogeneity between those identified as putative orthologs or paralogs by Clan_Check, suggesting that this was also not an issue (supplementary fig. S16, Supplementary Material online). Finally, we found no significant difference (chi-squared = 3.309, df = 2, *P* value = 0.1912) in the distribution of the high level gene functional categories between those gene families identified as putative orthologs or paralogs by Clan_Check (supplementary fig. S17, Supplementary Material online), suggesting that the filter was not generating gene sets of biased function.

### Hidden Paralogy Disproportionally Affects Divergence Time Estimates in the Gymnophiona

**Fig. 3.**
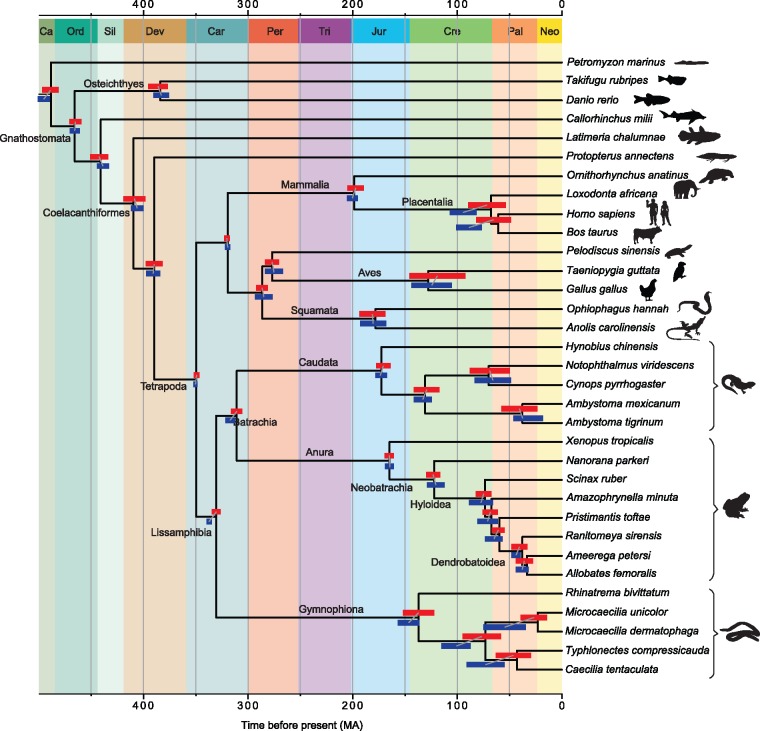
Timetree of vertebrates based on the most curated data set after enrichment for orthologs. Estimated upper and lower ranges for each node are represented as red/blue bars in each node. Red bars correspond to the divergence times estimated using Data set 348, the most curated data set. Dark blue bars correspond to the divergence times estimated using the larger Data set 768, which is before the “Clan_Check” step. Timescale in million years. Background colors for the geological time periods follow the International Commission on Stratigraphy.

We found that the inclusion of hidden paralogy in data sets affected divergence time estimates in specific clades of the Lissamphibia. When comparing timetree age estimates using Data set 768 (i.e., unfiltered) and Data set 348 (i.e., enriched in orthologs), we observed that divergence times for the internal nodes of the Gymnophiona appeared older with Data set 768 (Siphonopidae 55.89 Ma; Caeciliidae + Typhlonectidae 74 Ma; Divergence of Siphonopidae from Caeciliidae + Typhlonectidae 100.56 Ma) than with Data set 348 (Siphonopidae 23.12 Ma; Caeciliidae + Typhlonectidae 43.04 Ma; Divergence of Siphonopidae from Caeciliidae + Typhlonectidae 73.16 Ma) (fig. 3 and supplementary figs. S18 and S19 and supplementary tables S3 and S4, Supplementary Material online). Furthermore, Data set 768 resulted in older nonoverlapping confidence intervals for the divergence times of the Lissamphibia node compared with Data set 348 (with means of 337.03 and 330.41 Ma, respectively) (fig. 3). This trend was not observed within the Anura or Caudata where similar divergence time estimates were retrieved for both data sets. The older divergences inferred from data sets including putative paralogs is what would be expected given that the divergences of paralogs cannot postdate those of the taxa.

## Discussion

### Careful Outgroup Selection Allows for Simple but Effective Methods of Paralog Detection

We use an approach for detecting orthologs that involves the identification of carefully chosen outgroups. This allows the inclusion of incontestable outgroup relationships that should exist if the species are present in a gene family (see Materials and Methods for more details). For example, in our study of the Lissamphibian phylogenetic relationships, the mammals were treated as such an incontestable clan, which if violated, resulted in the removal of the gene family. We applied this rationale using several well-defined vertebrate relationships. Although this approach does not guarantee to filter all paralogous gene families, or indeed identify all orthologs (as patterns of late-gene loss can result in paralogs supporting all three possible topologies), it does offer a simple but effective method to enrich for orthologous gene families in a data set and increases overall support for the true topology. In addition to that, when using this approach we recommend that the ingroup taxa always outnumber the outgroup taxa to ensure that model optimization does not become overly biased away from the ingroup species being studied. In our study, only 15 of the 33 taxa were outgroups.

### Congruence among Methods Was Only Achieved with Enrichment for Orthologs

A surprising result of our analyses was the level of variation exhibited by each of the three methods used to reconstruct the phylogeny of the Lissamphibia. Two of the methods clearly demonstrated sufficient sensitivity toward the inclusion of paralogous relationships (ML and QS) as to yield the Procera hypothesis, with the Batrachia hypothesis found only after enriching for orthologs. In contrast, the stability of the results for the root of the Lissamphibia with BI (with the CAT-GTR model) across all the data sets suggests that such complex models are better able to discern the majority support in a data set, even when the difference in signal is small (which is the case in this data set, as demonstrated by the results of the gene-level AU tests). However, this stability does not extend to the more recent nodes within the Anura (supplementary figs. S2–S13, Supplementary Material online), something observed across several previous studies (Frost et al. 2006; Roelants et al. 2007; Pyron and Wiens 2011; Zhang et al. 2013; Feng et al. 2017; Heinicke et al. 2018; Streicher et al. 2018) and that could be from other sources of incongruence such as ILS. The lack of resolution at nodes within the Anura even when using large-scale data sets is an issue that merits further study with a data set better suited to addressing this problem than used here. Similarly, despite our consistent retrieval of turtles as a sister group to birds (fig. 3 and supplementary figs. S2–S13, Supplementary Material online), we cannot make a definite statement about this in the context of the vertebrate tree of life because the data set was not constructed to address this contentious phylogenetic issue (i.e., due to the absence of crocodiles from our data set).

Complex models in BI require access to substantial computational resources for large data sets (we required access to 64 CPUs for over a month for each chain on a national high-performance cluster before convergence was reached). Nevertheless, we find that such substantial requirements may be overcome with careful gene-family selection, as demonstrated by the convergence of all three methods to the same topology (Batrachia) when using Data set 348. These and the results of the gene-family AU tests confirm our hypothesis that paralogy drives support for alternative topologies in the Lissamphibia.

### Hidden Paralog Inclusion Impacts Divergence Time Estimates in the Lissamphibia

Considering the conflicting topologies retrieved for the root of the Lissamphibia as a result of paralog inclusion, the observed impact on their divergence time estimates may be expected. However, a surprising result was the impact of paralog inclusion on divergence time estimates for groups (such as the Gymnophiona) where all data sets and methods agreed on the same topology (fig. 3). Within the Gymnophiona more ancient divergence time estimates were retrieved when paralogs were included (Data set 768) than when they were removed (Data set 348) (fig. 3), a trend that extended to the Anura with the largest paralog-containing data set (Data set 2656, see supplementary fig. S20 and supplementary table S5, Supplementary Material online). Our most curated data set (Data set 348) concurs with recent estimates of the divergence times of Lissamphibia (Zhang et al. 2005; Okajima and Kumazawa 2009; Pyron 2010; Irisarri et al. 2017), Batrachia (Zhang et al. 2005; Pyron 2010; Irisarri et al. 2017), and its three extant lineages: Anura (Hugall et al. 2007; San Mauro 2010), Caudata (Zhang and Wake 2009; Kurabayashi et al. 2012; Bonett et al. 2013; Irisarri et al. 2017), and Gymnophiona (Pyron 2010; Irisarri et al. 2017).

### Conservative Ortholog Selection Should Be a Priority in Phylogenomic Analyses

Phylogenomic methods rely on large numbers of phylogenetically informative sites to produce accurate reconstructions risking the inadvertent inclusion of hidden paralogs from ancient duplication events. As shown, this can result in the retrieval of highly supported alternative topologies and it can have large effects on subsequent time estimates. This is equally a danger for concatenation and supertree approaches. Therefore, implementing robust approaches for filtering paralogs from genome-scale data sets should be a standard part of every phylogenomic pipeline. Achieving equilibrium between the opposing demands of large-scale versus quality can be difficult and failing to do so likely explains the historical conflicting relationships for the Lissamphibia with molecular data and is likely to apply to other groups with controversial topologies.

## Materials and Methods

### Taxon Sampling

In total, our analyses included 33 species: 18 ingroup species (amphibians) and 15 outgroups (other nonamphibian vertebrate species). The ingroup included eight anuran species, five salamanders and five caecilians (the complete list of species included is summarized in supplementary table S1, Supplementary Material online). The anuran species represented seven families: Aromobatidae, Bufonidae, Dendrobatidae, Dicroglossidae, Hylidae, Craugastoridae, and Pipidae; the caudatan species represented three families: Hynobiidae, Ambystomatidae, and Salamandridae; and the caecilian species represented four families: Caeciliidae, Rhinatrematidae, Siphonopidae, and Typhlonectidae. Six of the anuran species were represented by novel transcriptomic data generated for this study, whereas the two other (*Xenopus tropicalis* and *Nanorana parkeri*) had completely sequenced genomes available from Ensembl (http://www.ensembl.org/; last accessed June 1, 2016) and GigaScience Database (Sun et al. 2015), respectively. The five species of Caudata were chosen based on the quality and source of the transcriptomic data available in the NCBI database (https://www.ncbi.nlm.nih.gov/; last accessed June 10, 2016), favoring those with higher number of reads. Remarkably, for *Ambystoma mexicanum* and *Ambystoma tigrinum*, available transcriptomic data were scarce with only either expressed sequence tags or data sets targeting embryonic tissues or particular chromosomes available. In these cases, we combined all the different sources available to increase the pool of gene families for those species. A complete list of the accession numbers of data included can be found in supplementary table S1, Supplementary Material online. The five species of caecilians included were all those generated by Torres-Sánchez et al. (2019), which consisted of transcriptomic data from a selection of tissues for each of the five species examined (supplementary table S1, Supplementary Material online). Two of the species of caecilians (*Microcaecilia unicolor* and *Rhinatrema bivittatum*) were each represented by transcriptomes from two individuals. These were treated separately for the assembly, CDS prediction and orthology prediction stages at which point duplicate genes were removed. Additionally, we included 15 vertebrate taxa as outgroups, which included the lamprey, elephant shark, zebrafish, pufferfish, lungfish, coelacanth, king cobra, *Anolis* lizard, Chinese turtle, chicken, zebrafinch, platypus, elephant, cow, and human. We downloaded the CDS of the genomes for most of the species from the Ensembl database. The exceptions were the elephant shark, the lungfish, and the king cobra downloaded from NCBI and the elephant shark genome repository (see supplementary table S1, Supplementary Material online). All sequences were downloaded in April and August 2016.

### RNA Extraction and Sequencing

Live specimens of the following six species were collected in the Amazon rainforest of Eastern Peru during the rainy season (January 2015): *Amazophrynella minuta*, *Allobates femoralis*, *Ameerega petersi*, *Ranitomeya sirensis*, *Scinax ruber*, and *Pristimantis toftae*. Four replicates were collected per each species. All 24 specimens were anesthetized with 8% Benzocaine and euthanized with MS222. Samples of liver and skin were immediately taken from the specimens and fixed in RNAlater Solution (Ambion), following 24 h at room temperature, 12 days at −20 °C and finally long-term storage at −80 °C. The remainder of the carcasses were fixed in 10% formaldehyde following methods described in Heyer et al. (1994), rinsed in water, and finally stored in 70% ethanol and deposited at the Herpetology Collection of CORBIDI in Lima, Peru.

Total mRNA was extracted from the samples using the QIAGEN miRNAeasy kit following the manufacturer’s protocol. Quantity and quality of the extracted total mRNA was assessed with the NanoDrop ND-1000 spectrophotometer (Thermo Scientific) and Experion Labchip electrophoresis (BIORAD). Illumina Tru-Seq libraries for polyA-tail mRNA were prepared following manufacturer’s guidelines. All samples were sequenced on an Illumina HiSeq 2500 sequencer at the Translational Genomics facility in IBERS, Aberystwyth University.

### Bioinformatic Data Preprocessing

The pipeline used for obtaining the data set ready for phylogenomic analyses is summarized in supplementary fig. S21, Supplementary Material online. Raw reads obtained from the sequencer and SRA files downloaded from NCBI were processed for quality, read size and read number using FastQC v0.11.2 (Babraham Bioinformatics) and Trimmomatic (Bolger et al. 2014) (including adapter trimming). De novo transcriptome assemblies were generated for each species by combining all trimmed reads for all specimens and tissues per species into a single species assembly using Trinity v2.0.6 (Grabherr et al. 2011). Then, candidate coding genes were predicted from the transcriptome assembly with Transdecoder v2.0.1 (Haas et al. 2013). Given that some transcript predictions can give multiple isoforms of the same gene due to alternative splicing or heterogeneous alleles, the candidate coding genes were filtered by their longest open reading frame sequence. The amino acid translations of the resulting sets of filtered protein-coding genes constituted the input for the orthology prediction step (see supplementary fig. S21, Supplementary Material online). CDS downloaded from Ensembl or other sources (i.e., NCBI, GigaScience) were translated into amino acids, filtered for redundancies and presence of stop codons in the middle of the translated amino acid sequence with a custom bash script.

### Orthology Prediction

The identification of orthologous gene families is a key step for robust phylogenetic analyses. Since most of the amphibian species lack a reference genome (only present for *Xenopus tropicalis* and *Nanorana parkeri* when analyses were carried out), we wanted to use an approach that would allow for the identification of novel gene families that could be unique to the Amphibia. We carried out ortholog prediction with the OrthoMCL v2.0.9 pipeline (Li et al. 2003). Reciprocal all-against-all sequence similarity searches were carried out with Diamond v.0.7.9 (Buchfink et al. 2015) with a BIT-score cut-off of 60. The clustering steps in orthoMCL were set up with the default granularity (*I* = 1.5). As this resulted in a larger number of clusters than expected (i.e., 117,282 gene families, overestimating the number of gene families), we repeated the entire process using a hierarchical approach. This involved choosing a representative of each of the clusters obtained and carrying out another round of reciprocal sequence similarity searches among these and repeating the MCL clustering step (with granularity *I* = 2.0). This reduced the number of predicted clusters by more than half (resulting in 52,852 gene families), while also reducing the number of clusters represented by very few of taxa, and in turn increasing the number of clusters with representatives from all species (a visual summary of these steps can be seen in supplementary fig. S21, Supplementary Material online).

In order to separate single-copy from multicopy gene families, we used a tree-based approach that involved alignment with MUSCLE with default settings (Edgar 2004) and ML phylogeny reconstruction with RAxML v.8 (Stamatakis 2014) using a PROTGAMMAJTT model for all 52,852 gene families. Any remaining redundant gene transcripts were identified as species-specific clades in the resulting trees and only the longest representative of each was retained using the “prune-monophyly” command in CLANN v4.2 (Creevey and McInerney 2005). This approach also removes any species-specific gene duplicates (i.e., in-paralogs sensu Sonnhammer and Koonin 2002). Reduction of these species-specific duplicates to a single copy allows identification of gene families that would otherwise have been erroneously excluded for being in multiple copy. As the pruned sequences clustered together at the tips of the tree, the choice of representative does not affect the reconstruction of the species relationships being examined, however choosing the longest reduced the possibility of including partially reconstructed sequences in the analysis. Finally, we identified the single-copy gene families in the gene trees using the command “deletetrees multicopy” in CLANN v4.2. This resulted in 2,696 single-copy gene families.

### Data for Comparative Phylogenomics

We repeated the multiple sequence alignment step for the 2,696 gene families using a finer comparative approach (AQUA, Muller et al. 2010) which generates multiple alignments for each with different software tools (MUSCLE, Edgar 2004; MAFFT v7.3, Katoh and Standley 2013) with refinement in RASCAL (Thompson et al. 2003) and chooses the best based on a normalized score implemented in NORMD (Thompson et al. 2001). Poorly aligned regions were then removed using Gblocks (Castresana 2000) with “relaxed” parameter settings (max. number for large number of contiguous nonconserved positions: 50; minimum number of sequences for flank position: 50%; minimum length of a block: 2; allowing all gap positions). Using these alignments, we assessed each of the 2,696 gene families for sequence saturation at the amino acid level by calculating for each the sum of all branch lengths of Neighbor-Joining phylogenetic trees constructed under p-distance or JTT models of evolution in PHYLIP (Felsenstein 1989). The p-distance and JTT model distances of every tree were plotted against each other in a single graph and examined by eye for outliers and signatures of saturation (where the sum of branch lengths calculated with the JTT model which accounts for multiple substitutions was substantially greater than that calculated using the p-distance). This step identified 40 gene families for removal reducing the data set to 2,656 gene families. The best evolutionary model for each of the remaining gene families was estimated with ModelGenerator (Keane et al. 2006) and gene trees were inferred again using RAxML, this time using the best-fitting models predicted for each gene family with 100 bootstrap replicates. This resulted in the 2,656 alignments, models, and gene trees that were used for the subsequent analyses.

### Enriching for Orthologs with Clan_Check

The aim of the present study was to test the hypothesis that hidden paralogy as a result of ancient genome (or gene) duplications could be driving highly supported alternative topologies at the root of the Lissamphibia. This is based on the premise that some of the single-copy gene families in extant jawed vertebrates may violate the assumption of deep orthology due to ancient duplications events that occurred prior to the LCA of this group (fig. 1*B*). In this scenario, duplicate genes may be lost quickly, prior to any subsequent speciation events (defined as “early loss,” fig. 1*C*) or more slowly, possibly even following subsequent speciation events (defined as “late loss,” fig. 1*D*). The use of late-gene loss single-copy gene families to reconstruct the phylogenetic history of a group can result in paraphyletic reconstructions (i.e., violation of monophyletic clades, fig. 1*D*).

We used this knowledge to design a filtering approach that identified gene families that could not retrieve any *clans* (sensu Wilkinson et al. 2007) which were “incontestable” and should group together to the exclusion of the rest of the taxa (regardless of their internal splits). Using Clan_Check v.1.0 (https://github.com/ChrisCreevey/clan_check) and henceforth called the “Clan_Check” step, seven incontestable groups from the jawed vertebrates were examined: mammals, birds, birds + reptiles, frogs, salamanders, caecilians, and tetrapods (see supplementary table S1, Supplementary Material online, for more details), where for each, all members present in the gene family must be found together in a clan. Each of these incontestable clans was chosen based on the availability of genomic or transcriptomic data (supplementary table S1, Supplementary Material online) and on scientific consensus that they should group together in a clan to the exclusion of all other taxa in the study (without making any assumptions about the interrelationships within or between these groups). The Clan_Check approach is based on the hypothesis that late-gene loss following an ancient duplication event would result in paralogy in multiple descendent lineages (fig. 1*D*) and so identification of gene families where incontestable clans are violated should enrich the data set for genes supporting the true topology. Even though in the 4-taxon case, paralogy caused by late-gene loss is indistinguishable from ILS, our approach is insensitive to ILS. This is because ILS is lineage-specific and there is no reason to expect that an increase in ILS in one vertebrate lineage would be related to an increase in ILS in another distantly related lineage. To demonstrate this, we used Simphy v1.0 (Mallo et al. 2016) to simulate 100 replicates of two data sets, the first generated with only ILS, and the second generated under a model of early duplications followed by ILS and late loss (generating paralogous single-copy gene families). See supplementary Methods, Supplementary Material online, for the control files and scripts used for this step.

### Data Sets for Testing

We generated four different data sets (each with alignments, models, and gene trees) to test the support for the relationships at the base of the Lissamphibia based on the following filters:

- Data set 2656: Containing all 2,656 gene families that resulted from the previous steps. This contained gene families ranging in size from 4 to 32 taxa, with an average of 10 taxa per gene family and 68% missing data.
- Data set 2019: Excluding gene families from the 2,656 data set that were not able to return expected monophylies in outgroup species following analysis with Clan_Check. This identified 637 gene families that were unable to reconstruct one or more of these “incontestable” clans and were removed. This resulted in a data set of 2,019 putative orthologs ranging in size from 4 to 32 taxa, with an average of 8 taxa per gene family and 76% missing data.
- Data set 348: Excluding putative orthologs based on data completeness. Using Data set 2019 as a starting point, we retained only those gene families containing at least 6 taxa with at least one anuran, one salamander, one caecilian and one outgroup included. The decision for including at least one of each of the amphibian groups was taken to be able to only include genes that could resolve our question of interest. This resulted in a reduction of the 2,019 ortholog data set to 348 orthologs ranging in size from 6 to 32 taxa, with an average of 18 taxa per gene family and 43% missing data.
- Data set 768: For comparison purposes, and to test the effect of the Clan_Check step, we carried out the same filtering step as in Data set 348 but using the Data set 2656 as a starting point (i.e., skipping the Clan_Check step). This resulted in a reduction of the 2,656 data set to 768 gene families ranging in size from 6 to 32 taxa, with an average of 20 taxa per gene family and 36% missing data.

### Phylogenomic Analyses

To understand the effect of phylogenetic method choice on resulting phylogenies from the different data sets, we used two supermatrix methods: ML and BI; and a QS method, that can be consistent in the face of ILS (Mirarab and Warnow 2015). Each of the four data sets (Data set 2656, Data set 2019, Data set 348, and Data set 768) was analyzed with all three methods. For the supermatrix approaches, we concatenated the alignments for each data set and inferred ML trees with 100 bootstraps in the parallel version of RAxML-Pthreads (Stamatakis 2014) specifying the optimal model predicted earlier for each gene partition. The same concatenated matrices were then used to obtain phylogenetic trees with BI using Phylobayes-MPI v.1.7 (Rodrigue and Lartillot 2014) under the CAT-GTR model (with the options “-cat -gtr”), which assumes that the rate across sites is under a discrete gamma (four categories). We ran two chains for each of the four data sets until convergence was reached defined as when the mean difference as calculated by “bpcomp” between the chains was <0.03 using a sampling frequency of 10. Convergence was observed for Data set 2656 following 1,230 and 1,158 cycles for the first and second chain respectively resulting in a max diff of 0 and a mean diff of 0 after discarding the initial 200 as burn-in. Convergence was observed for Data set 2019 following 2,598 and 2,417 cycles for the first and second chain respectively resulting in a max diff of 0.021 and a mean diff of 0.0003 after discarding the initial 1,000 as burn-in. Convergence was observed for Data set 348 following 3,949 and 3,658 cycles for the first and second chain respectively resulting in a max diff of 0.067 and a mean diff of 0.001 after discarding the initial 1000 as burn-in. Finally, convergence was observed for Data set 768 following 892 and 908 cycles for the first and second chain respectively resulting in a max diff of 0 and a mean diff of 0 after discarding the initial 500 as burn-in. To check that these results were replicable, we repeated the analyses for Data sets 2656 and Data set 2019 and retrieved the same resulting topologies following convergences within a similar number of cycles.

For the supertree approach, we used the gene trees calculated for each of the gene families from the “Data sets for testing” section, and obtained a supertree with ASTRAL-II v.4.10.12 (Mirarab and Warnow 2015) for each data set. For a schematic summary of the phylogenomic analyses carried out, see supplementary fig. S21, Supplementary Material online. All computational analyses were carried out using the Aberystwyth University HPC servers and High-Performance Computing clusters in Wales (HPC Wales), using up to 64 cores for a period of 6 weeks for the largest data sets.

### Testing the Gene-Family Support for the Three Possible Topologies at the Base of the Lissamphibia

In order to examine the gene-level support for each of the three possible topologies at the base of the Lissamphibia we carried out AU tests for all 768 gene families that were capable of addressing this question (which also included the putative ortholog subset, Data set 348). The individual gene-family alignments generated earlier with AQUA were used as input to RAxML v.8 (Stamatakis 2014) with their best predicted model (estimated as outlined earlier) in order to generate site-likelihoods using the “‐f G” option (which allowed the model parameters to be re-estimated for each tree). The three trees provided via the “-z” option were individually pruned to the taxa-set specific to each gene family. The resulting site-likelihoods for each of the 768 gene families were then provided as input to CONSEL (Shimodaira and Hasegawa 2001) first using the command “makermt –puzzle” to generate bootstrap replicates, followed by “consel” to calculate the statistics and finally “catpv” to print the resulting *P* values. A summary of the results can be found in supplementary table S2, Supplementary Material online.

### Testing for Long Branch Attraction, Compositional Heterogeneity, and Gene Ontology Bias

We used Data sets 768 and its subdata set 348 to test whether the Clan_Check filter could be biased toward branch length, compositional heterogeneity or gene function. These data sets were chosen as they were the only ones that had the taxon coverage to address the Lissamphibia question (i.e., had to have presence of at least one Anura species, one Caudata species, one Gymnophiona species, and one outgroup) and have a minimum of six taxa. To test if there was a difference in the distribution of average branch lengths in those gene families identified as putative orthologs or paralogs by Clan_Check, we calculated the average branch length per gene tree (constructed earlier) for each of the 768 gene trees by summing the branch lengths and dividing by the number of (internal and external) branches on the tree. We then tested with a Wilcoxon Signed-Rank test in R (R Core Team 2016) the null hypothesis that there was no difference in the average branch lengths in the set of gene classified as either putative orthologs or paralogs by Clan_Check. To test whether Clan-Check could be biased for taxa with compositional heterogeneity issues, we obtained the proportion of taxa that failed the Compositional Heterogeneity test from running each of the 768 gene tree alignments in IQ-TREE (Nguyen et al. 2015) specifying its own best model (as obtained from ModelGenerator), and we retrieved the number of failed taxa, which we divided by the total number of taxa in each alignment. As in the branch length test, we performed a Wilcoxon Signed-Rank test in R, the null hypothesis being that there was no difference in the proportion of taxa that failed compositional heterogeneity in the set of genes classified as either putative orthologs or paralogs by Clan_Check. Finally, to test whether Clan_Check was biased for genes which could have a particular type of gene function, we obtained functional annotations (GOs) for all 768 genes from Blast2GO v5.2 (https://www.blast2go.com/; last accessed September 21, 2018) installed on a local computer. GOs were then grouped into their three major categories: “Cellular component,” “Molecular function,” and “Biological process” and counted. We performed a Pearson’s chi-square test (test of independence) between putative orthologs and paralogs as classified by Clan_Check using R.

### Time Tree Estimations

Finally, we carried out a time tree analysis using the different data sets and trees constructed. This involved using an auto-correlated log-normal relaxed clock model with sequence evolution model CAT-GTR in Phylobayes v.4.1 (Lartillot and Philippe 2004). We used a birth–death prior on divergence times for noncalibrated nodes and included 12 calibration points taken from the Fossil Calibration Database (http://fossilcalibrations.org/; last accessed January 30, 2018), a carefully curated database that uses verified fossil records from the literature (Ksepka et al. 2015) and treated all calibrations as soft boundaries. A summary of the calibrated nodes is available in supplementary table S6, Supplementary Material online. Using these settings, we tested the effect of data set size and paralog inclusion by estimating divergence times with the 768 Data set and the 348 Data set (which recovered the same topology with BI). A further timetree was also estimated using Data set 2656. For all analyses, Phylobayes was run until reaching 10,000 data points sampling every 10 after a burn-in of 5,000.

## Supplementary Material

Supplementary data are available at *Molecular Biology and Evolution* online.

## Supplementary Material

Supplementary_Material_msz067
