# Supplementary material for: Inadvertent Paralog Inclusion Drives Artifactual Topologies and Timetree Estimates in Phylogenomics
Source: Mol Biol Evol. 2019 Mar 23;36(6):1344–56. doi: 10.1093/molbev/msz067 (PMC6526904; doi:10.1093/molbev/msz067)
Supplement: Supplementary_Material_msz067 [file supplementary_material_msz067.pdf]

Supplementary Table S1. Details of all transcriptomic, genomic and EST sequences per species included in this study. In the assembled transcriptomes, number of contigs and N50 (in parentheses) is specified for both downloaded and *de novo* sequencing. For genomic data number of CDS downloaded is specified. Last column contains the source of the data. Species denoted with "*" were each represented by transcriptomes from two individuals (see Methods).

Class, Order and Family	Species names	Common name	Transcriptome Assembly: No. Contigs (N50)	ESTs	Genomic (# CDS)	# of CDS included after filtering for longest	Sources
**Amphibia**							
**Anura**							
Aromobatidae	*Allobates femoralis*	-	251,124 contigs (847)	-	-	50756	This study, GenBank: PRJNA430346
Bufonidae	*Amazophrynella minuta*	-	155,527 contigs (898)	-	-	36585	This study, GenBank: PRJNA430346
Craugastoridae	*Pristimantis toftae*	-	98,804 contigs (709)	-	-	25318	This study, GenBank: PRJNA430346
Dendrobatidae	*Ameerega petersi*	-	85,877 contigs (574)	-	-	19811	This study, GenBank: PRJNA430346
Dendrobatidae	*Ranitomeya sirensis*	-	368,483 contigs (754)	-	-	82092	This study, GenBank: PRJNA430346
Dicroglossidae	*Nanorana parkeri*	-	-	-	Genomic 22,972 protein coding sequences	23410	Predicted CDS downloaded from http://gigadb.org/dataset/100132
Hylidae	*Scinax ruber*	-	182,237 contigs (832)	-	-	42569	This study, GenBank: PRJNA430346
Pipidae	*Xenopus tropicalis*	-	-	-	Genomic 22,718 protein coding sequences	18429	Ensembl v84 / JGI 4.2
**Caudata**							
Ambystomatidae	*Ambystoma mexicanum*	-	173,592 contigs (902)	26,101	-	63475	SRR650441 - SRR650443; ESTs from NCBI with a Taxonomy search
Ambystomatidae	*Ambystoma tigrinum*	-	57,748 contigs (445)	20,495	-	21230	http://dx.doi.org/10.5061/dryad.49s43; EST ID: LIBEST_015526
Hynobiidae	*Hynobius chinensis*	-	146,142 contigs (804)	-	-	41938	SRR1042328
Salamandridae	*Cynops pyrrhogaster*	-	451,790 contigs (1509)	-	-	121567	SRR1051839
Salamandridae	*Notophthalmus viridescens*	-	56,401 contigs (1306)	-	-	34899	Assembly downloaded from http://newt-omics.mpi-bn.mpg.de/De_Novo_Transcriptome_Newt.fasta
**Gymnophiona**							
Caeciliidae	*Caecilia tentaculata*	-	287,446 contigs (1628)	-	-	72328	GenBank: PRJNA387587
Rhinatrematidae	*Rhinatrema bivittatum**	-	284,451 (1561); 276,470 (1519)	-	-	68390 and 73577	GenBank: PRJNA387587
Siphonopidae	*Microcaecilia unicolor**	-	166,567 (1569); 232,851 (1450)	-	-	45933 and 54630	GenBank: PRJNA387587
Siphonopidae	*Microcaecilia dermatophaga*	-	199,546 contigs (1574)	-	-	47965	GenBank: PRJNA387587
Typhlonectidae	*Typhlonectes compressicauda*	-	265,047 contigs (1454)	-	-	67739	GenBank: PRJNA387587
**Agnatha**							
	Petromyzon marinus	Lamprey	-	-	Genomic 11442 protein coding sequences	10,402	Ensembl v84 / Pmarinus_7.0
**Gnathostomes**							
	Callorhinchus milii	Elephant shark	-	-	Genomic 28158 protein coding sequences	31066	Predicted cDNA from http://esharkgenome.imcb.a-star.edu.sg/download/
	Danio rerio	Zebrafish	-	-	Genomic 50142 protein coding sequences	25571	Ensembl v84 / GRCz10
	Takifugu rubripes	Pufferfish	-	-	Genomic 47841 protein coding sequences	18510	Ensembl v84 / FUGU 4.0
	Protopterus annectens	Lungfish	154,496 contigs (1616)	-		40822	SRR505721–SRR505726, SRR088619–SRR088625
	Latimeria chalumnae	Coelacanth	-	-	Genomic 23601 protein coding sequences	19556	Ensembl v84 / LatCha1
**Reptilia**							
	Ophiophagus hannah	Cobra	-	-	Genomic 18,467 protein coding sequences	18467	GenBank: AZIM01155477.1
	Anolis carolinensis	Anolis	-	-	Genomic 19177 protein coding sequences	18585	Ensembl v84 / AnoCar2.0
	Pelodiscus sinensis	Turtle	-	-	Genomic 20669 protein coding sequences	18177	Ensembl v84 / PelSin_1.0
**Aves**							
	Gallus gallus	Chicken	-	-	Genomic 16354 protein coding sequences	15495	Ensembl v84 / Galgal4
	Taeniopygia guttata	Zebrafinch	-	-	Genomic 18204 protein coding sequences	17475	Ensembl v84 / taGut3.2.4
**Mammalia**							
	Ornithorhynchus anatinus	Platypus	-	-	Genomic 23584 protein coding sequences	21684	Ensembl v84 / OANA5
	Loxodonta africana	Elephant	-	-	Genomic 25635 protein coding sequences	20021	Ensembl v84 / Loxafr3.0
	Bos taurus	Cow	-	-	Genomic 22118 protein coding sequences	19982	Ensembl v84 / UMD3.1
	Homo sapiens	Human	-	-	Genomic 156921 protein coding sequences	22071	Ensembl v84 / GRCh38.p5

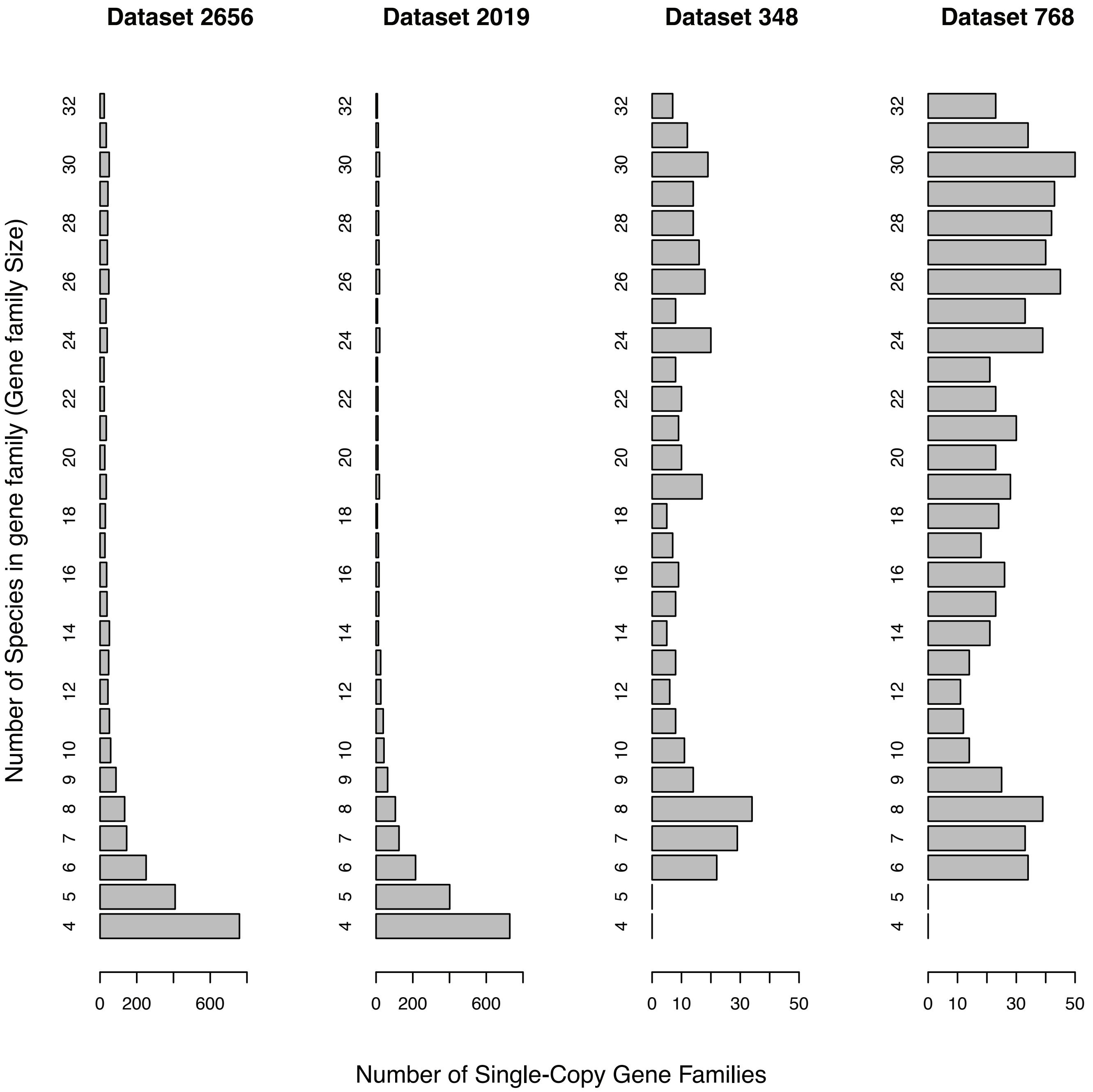

Supplementary Fig. S1. The size (number of species per gene family), distribution and number of gene families for each of the 4 datasets examined in this study.

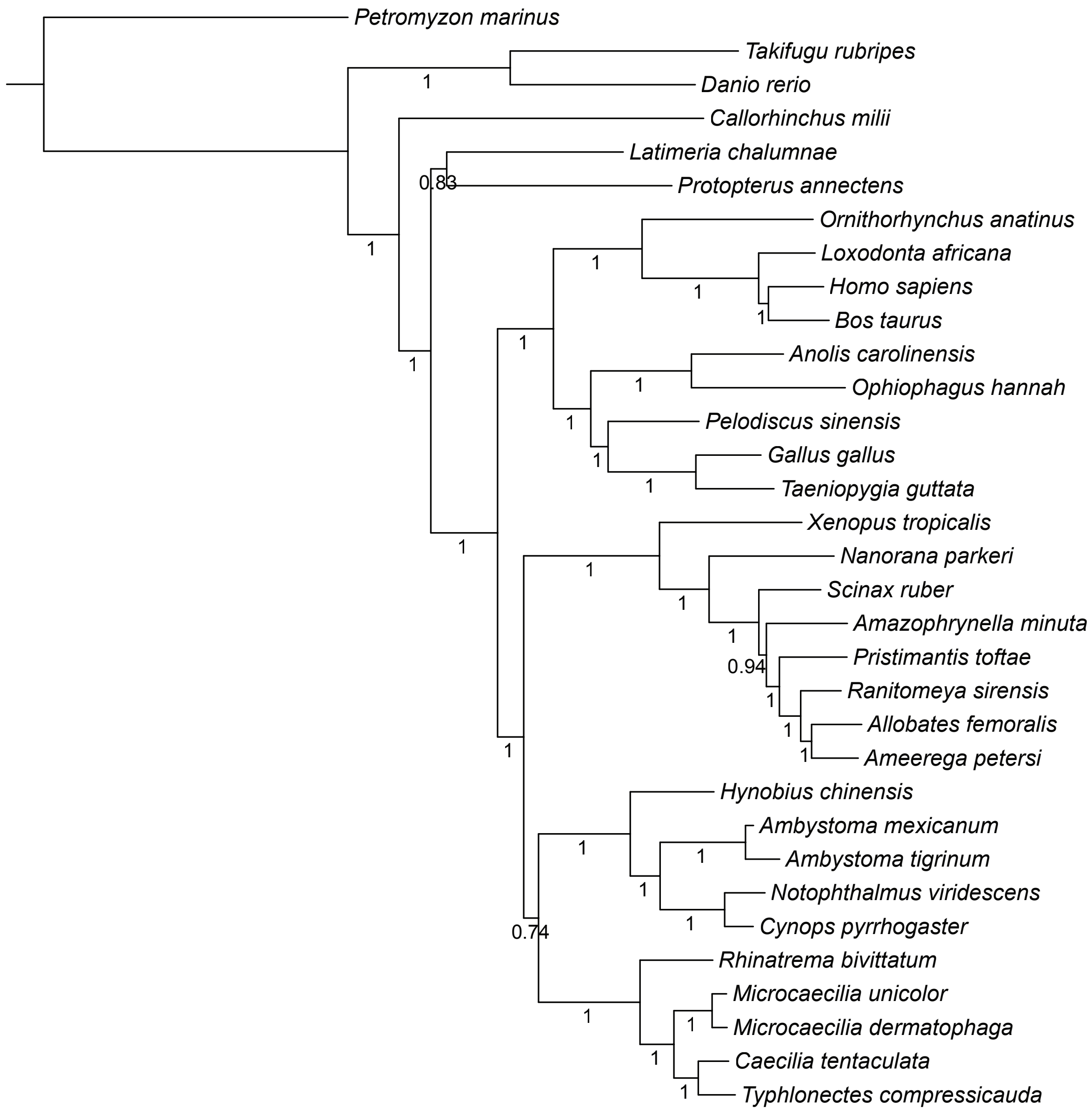

Supplementary Fig. S2. Resulting tree inferred with a concatenated alignment of Dataset 2656 using the Maximum Likelihood method in RAxML specifying individual models for each gene partition. Bootstrap support values provided under each node converted to a scale from 0-1, maximum support = 1. See methods for further details.

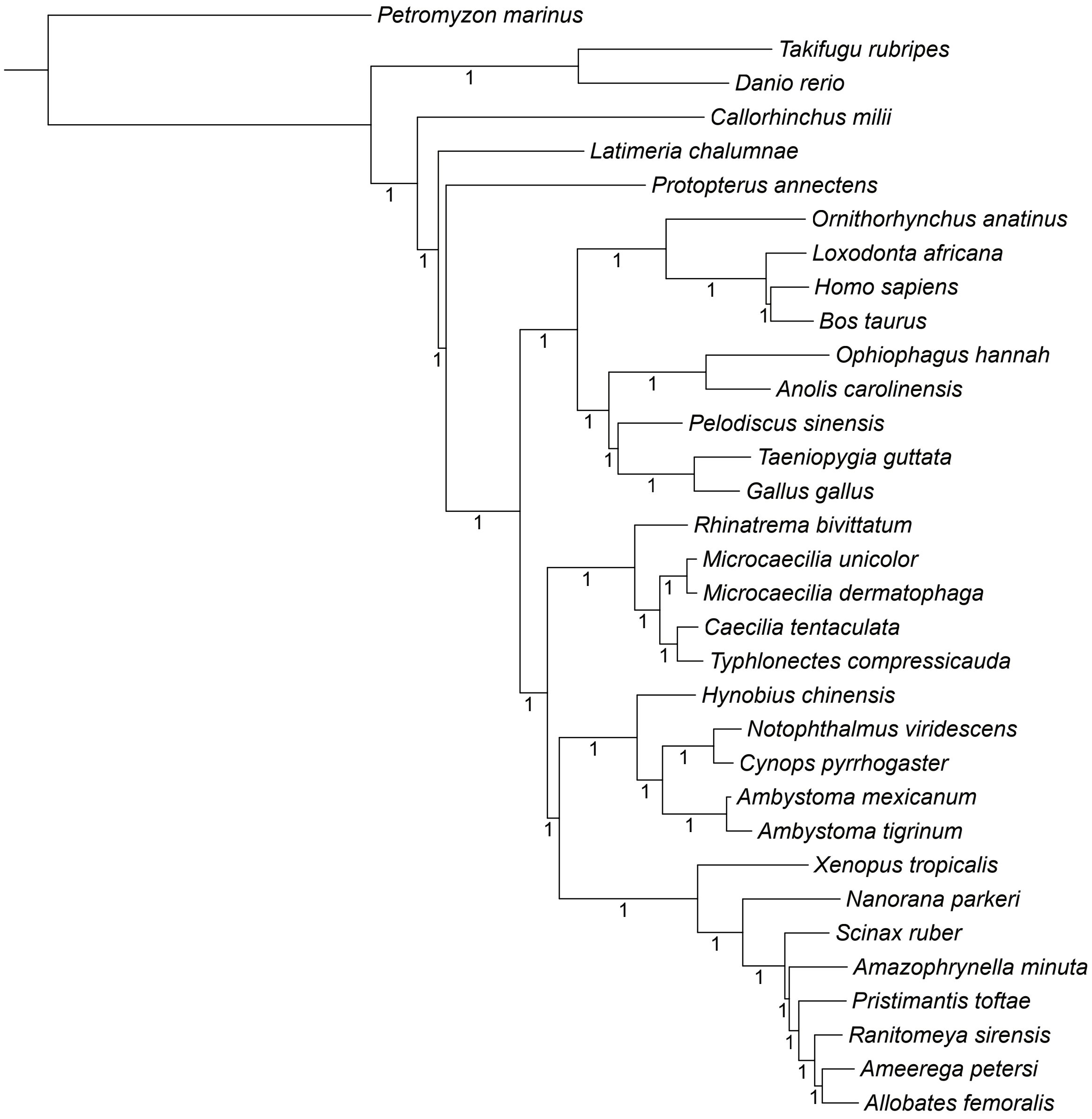

Supplementary Fig. S3. Resulting tree inferred with a concatenated alignment of Dataset 2656 using the Bayesian inference method in Phylobayes-MPI with the CAT-GTR model. Posterior probability support values are provided under each node, maximum support = 1. See methods for further details.

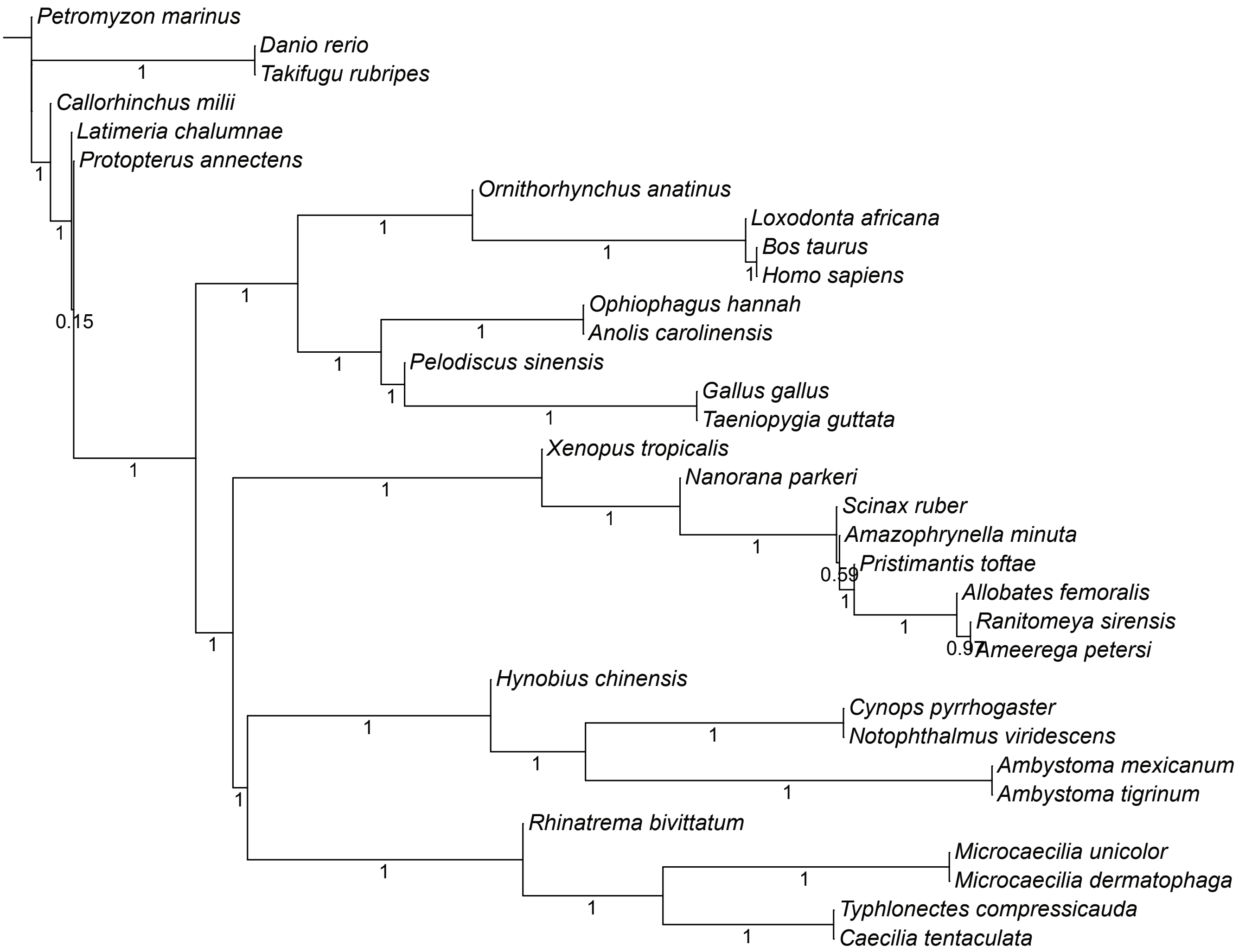

Supplementary Fig. S4. Resulting tree inferred with input gene trees from Dataset 2656 using the Quartet based supertree method ASTRAL-II. Local posterior probability support values are provided under each node, maximum support = 1. See methods for further details.

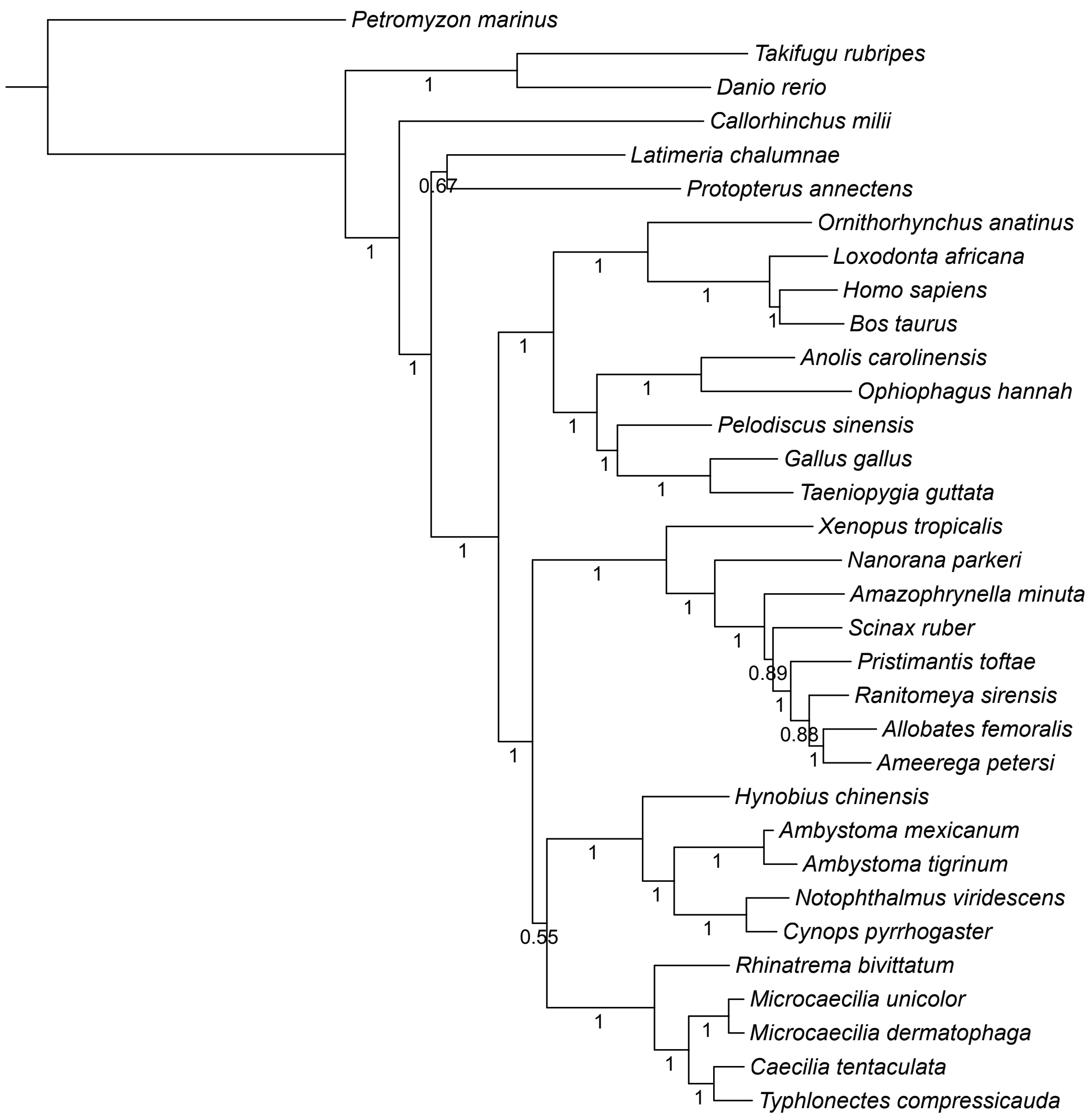

Supplementary Fig. S5. Resulting tree inferred with a concatenated alignment of Dataset 2019 using the Maximum Likelihood method in RAxML specifying individual models for each gene partition. Bootstrap support values provided under each node converted to a scale from 0-1, maximum support = 1. See methods for further details.

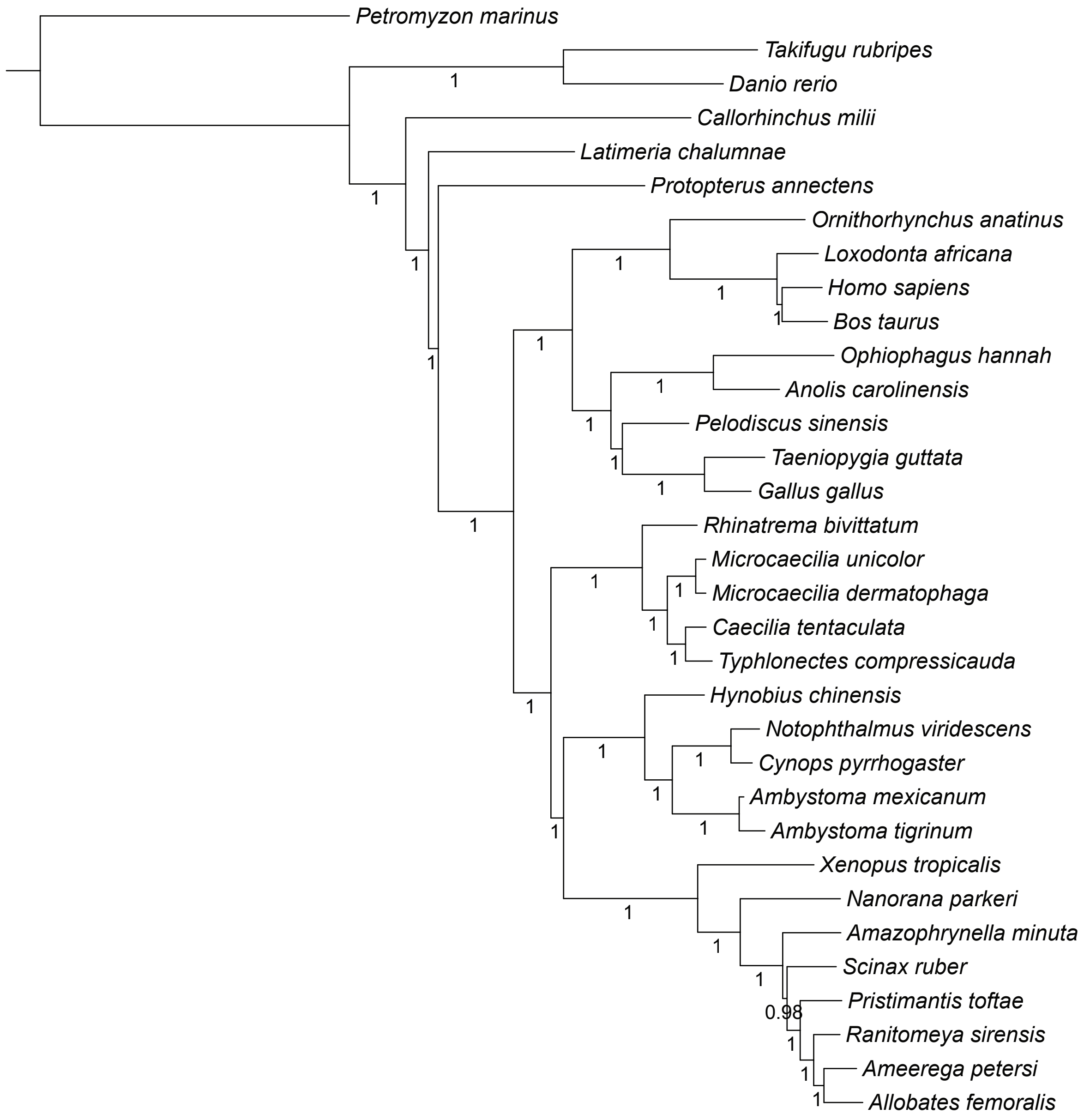

Supplementary Fig. S6. Resulting tree inferred with a concatenated alignment of Dataset 2019 using the Bayesian inference method in Phylobayes-MPI with the CAT-GTR model. Posterior probability support values are provided under each node, maximum support = 1. See methods for further details.

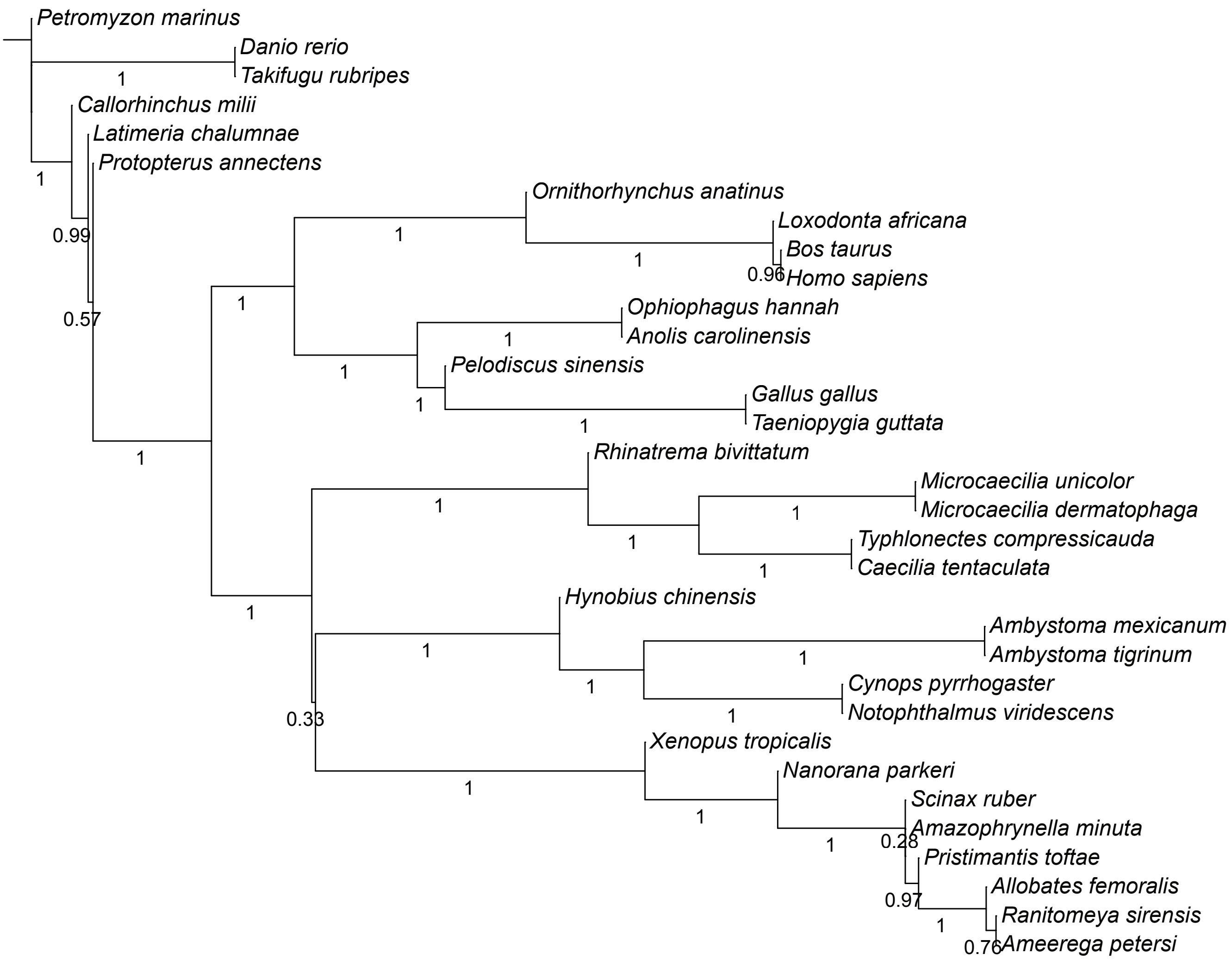

Supplementary Fig. S7. Resulting tree inferred with input gene trees from Dataset 2019 using the Quartet based supertree method ASTRAL-II. Local posterior probability support values are provided under each node, maximum support = 1. See methods for further details.

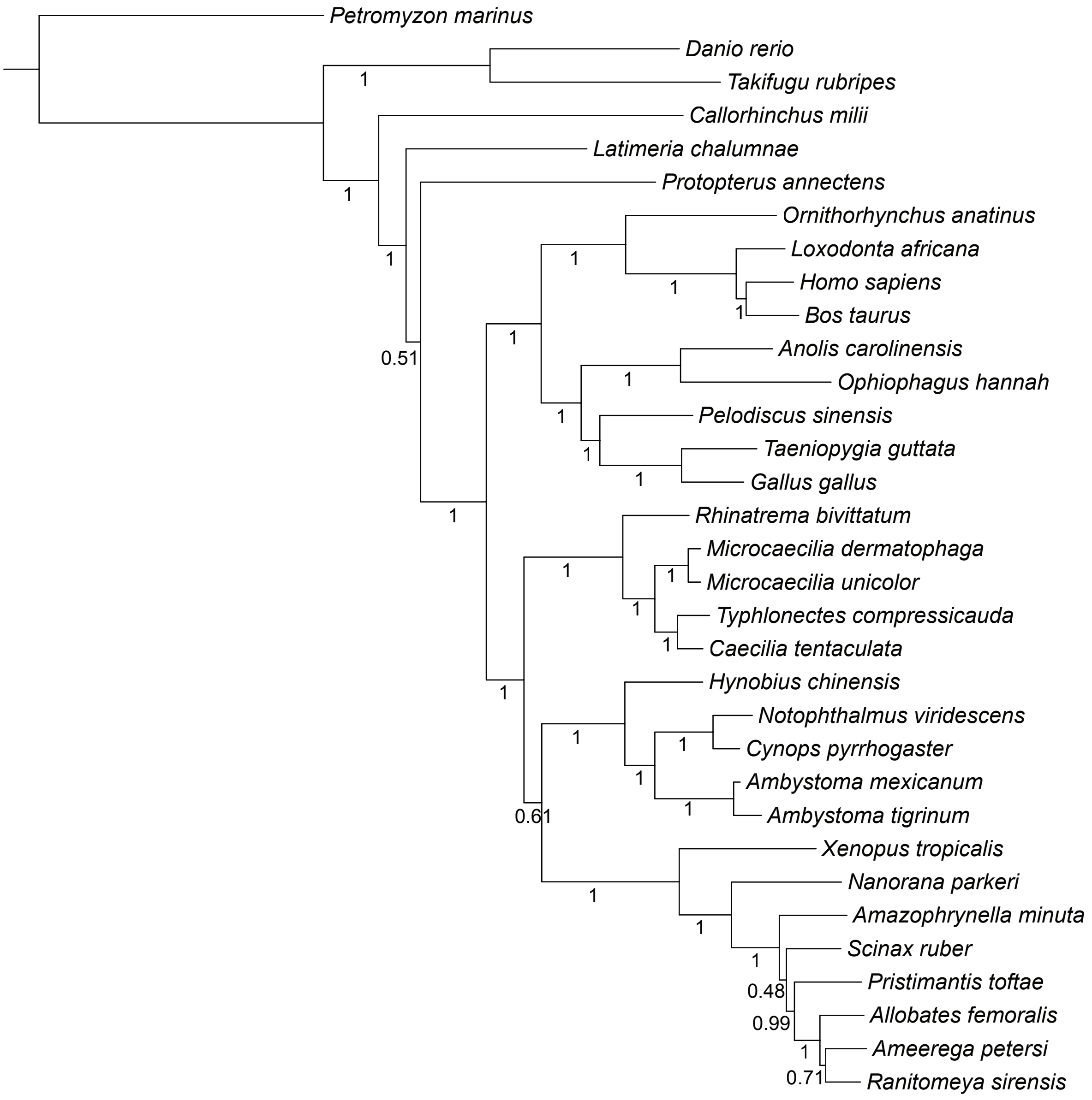

Supplementary Fig. S8. Resulting tree inferred with a concatenated alignment of Dataset 348 using the Maximum Likelihood method in RAxML specifying individual models for each gene partition. Bootstrap support values provided under each node converted to a scale from 0-1, maximum support = 1. See methods for further details.

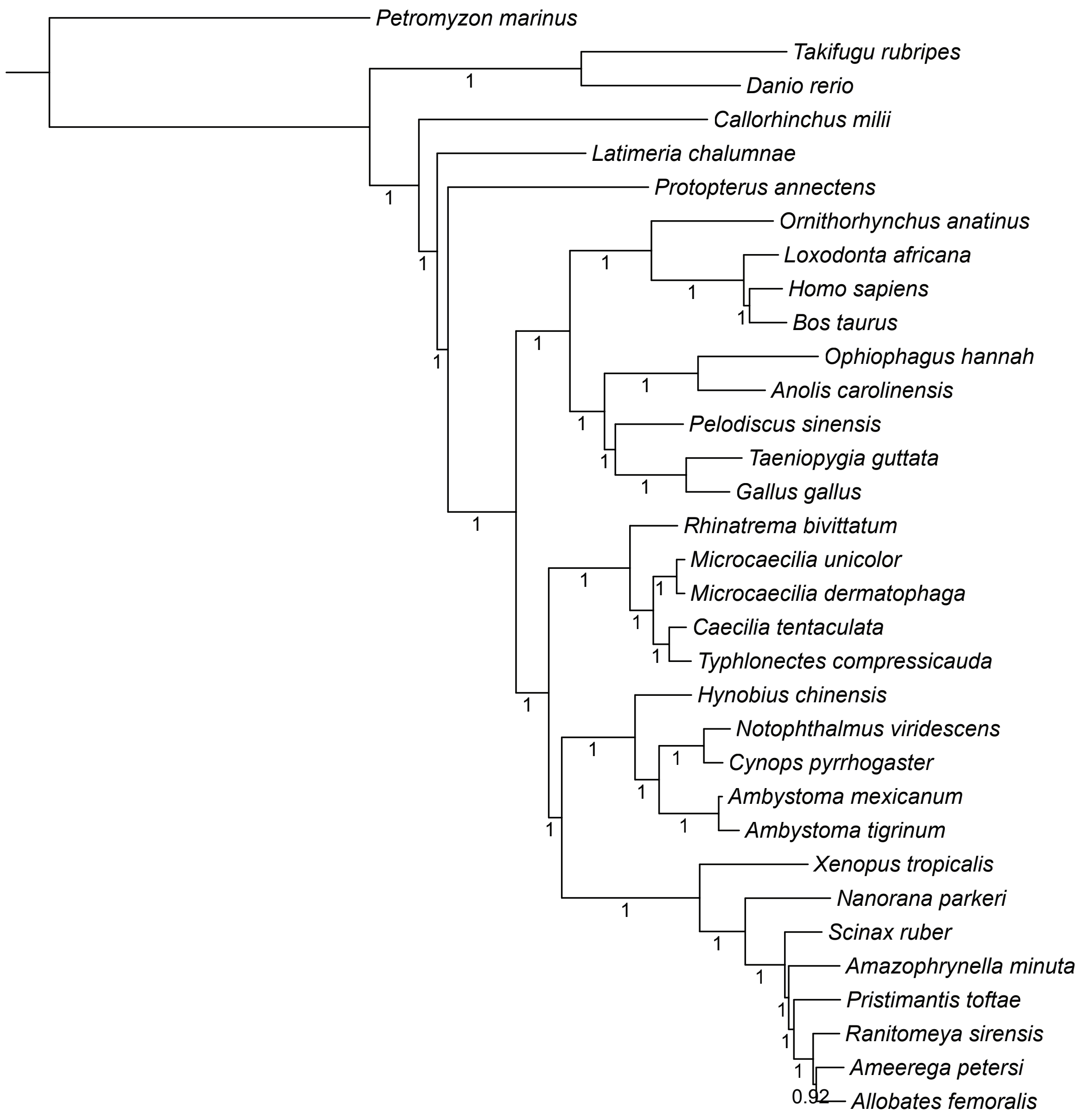

Supplementary Fig. S9. Resulting tree inferred with a concatenated alignment of Dataset 348 using the Bayesian inference method in Phylobayes-MPI with the CAT-GTR model. Posterior probability support values are provided under each node, maximum support = 1. See methods for further details.

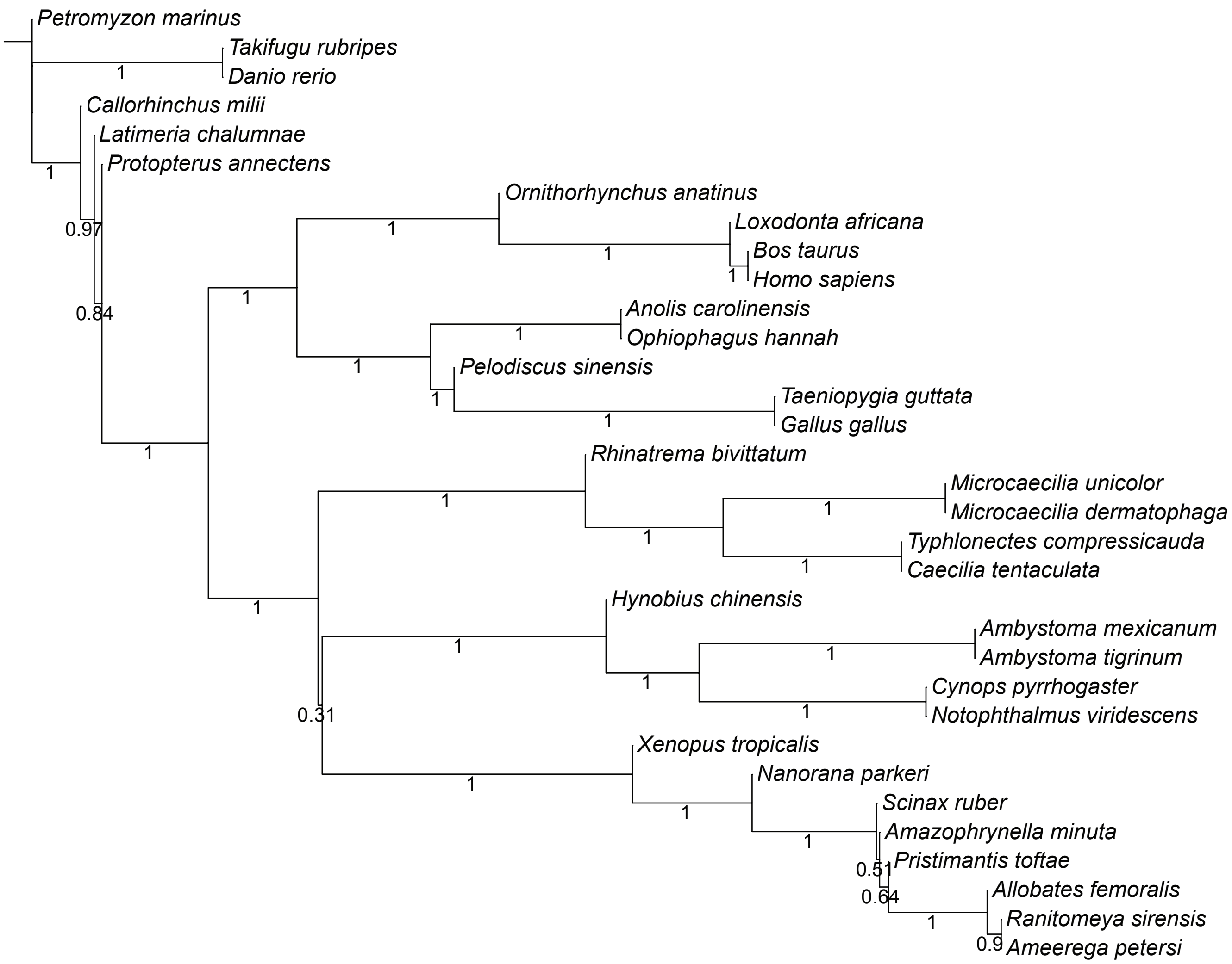

Supplementary Fig. S10. Resulting tree inferred with input gene trees from Dataset 348 using the Quartet based supertree method ASTRAL-II. Local posterior probability support values are provided under each node, maximum support = 1. See methods for further details.

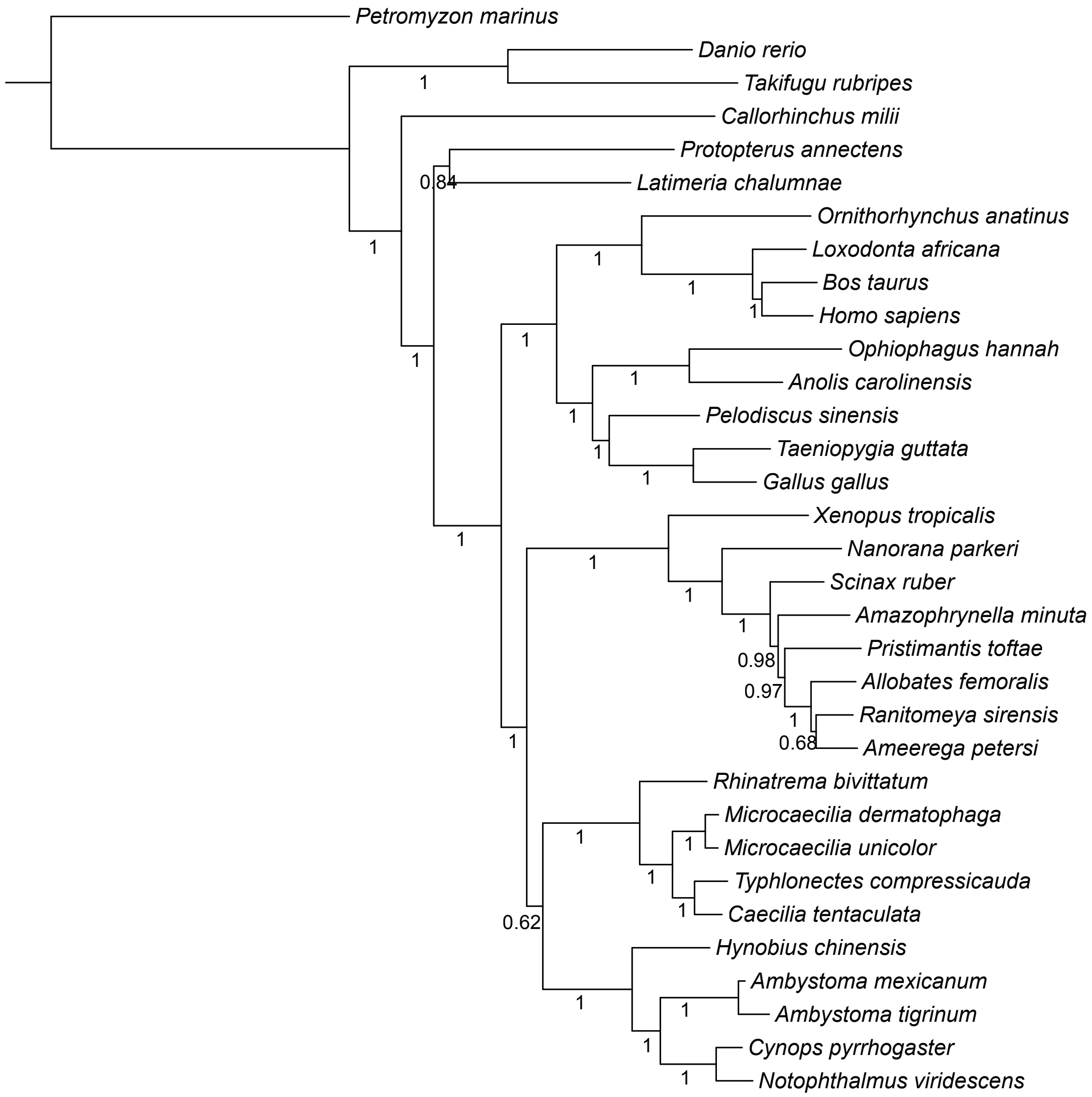

Supplementary Fig. S11. Resulting tree inferred with a concatenated alignment of Dataset 768 using the Maximum Likelihood method in RAxML specifying individual models for each gene partition. Bootstrap support values provided under each node converted to a scale from 0-1, maximum support = 1. See methods for further details.

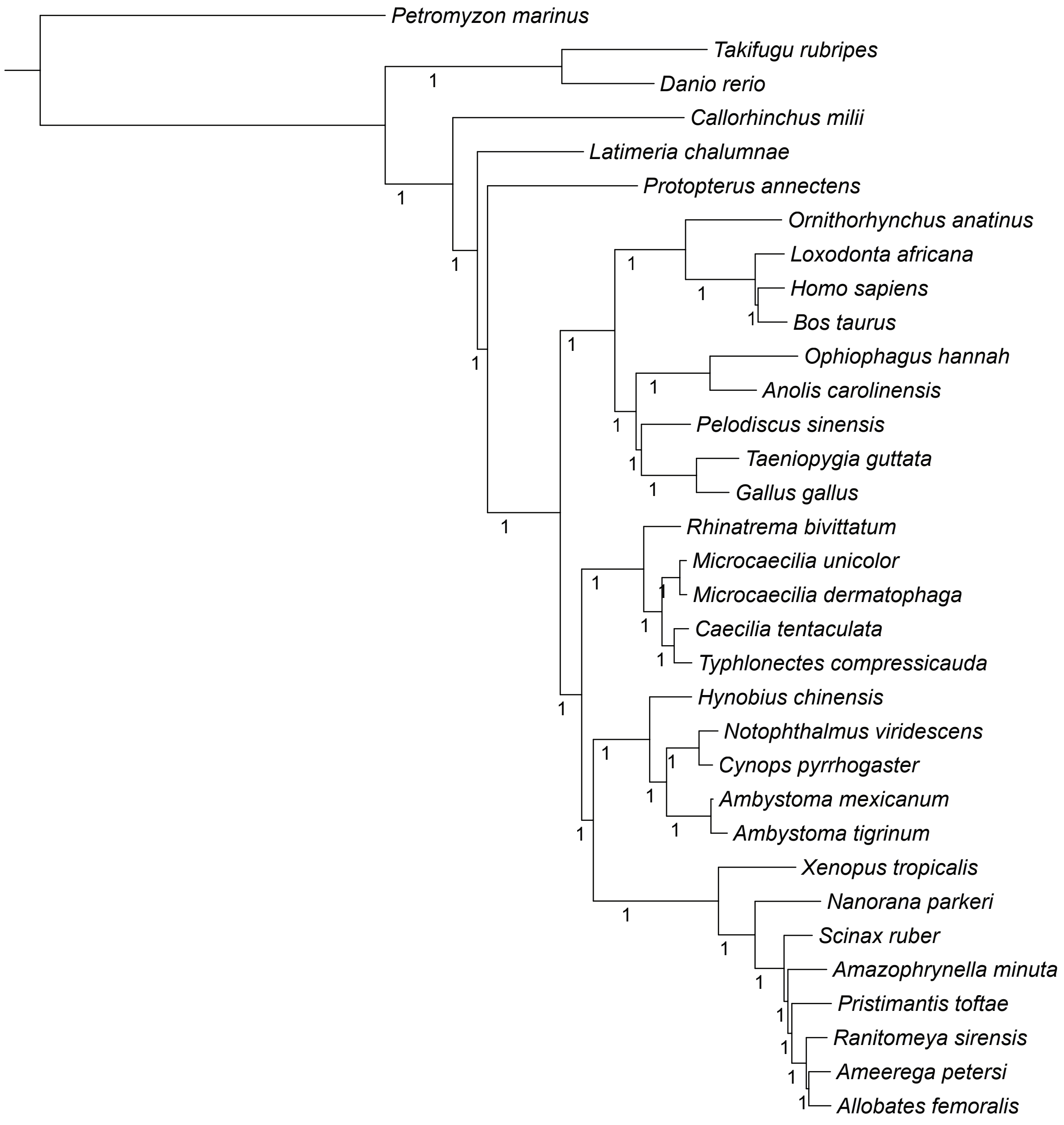

Supplementary Fig. S12. Resulting tree inferred with a concatenated alignment of Dataset 768 using the Bayesian inference method in Phylobayes-MPI with the CAT-GTR model. Posterior probability support values are provided under each node, maximum support = 1. See methods for further details.

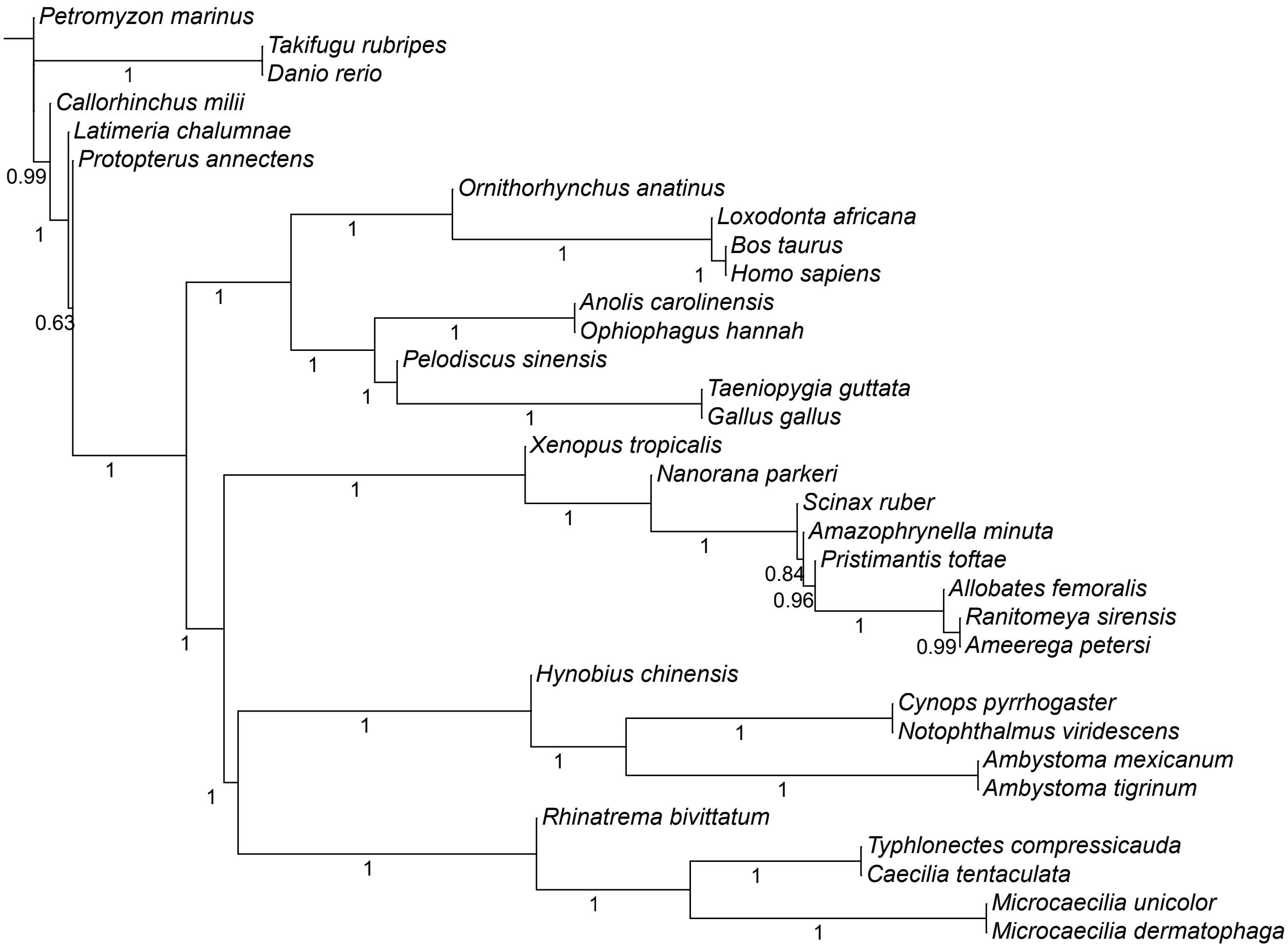

Supplementary Fig. S13. Resulting tree inferred with input gene trees from Dataset 768 using the Quartet based supertree method ASTRAL-II. Local posterior probability support values are provided under each node, maximum support = 1. See methods for further details.

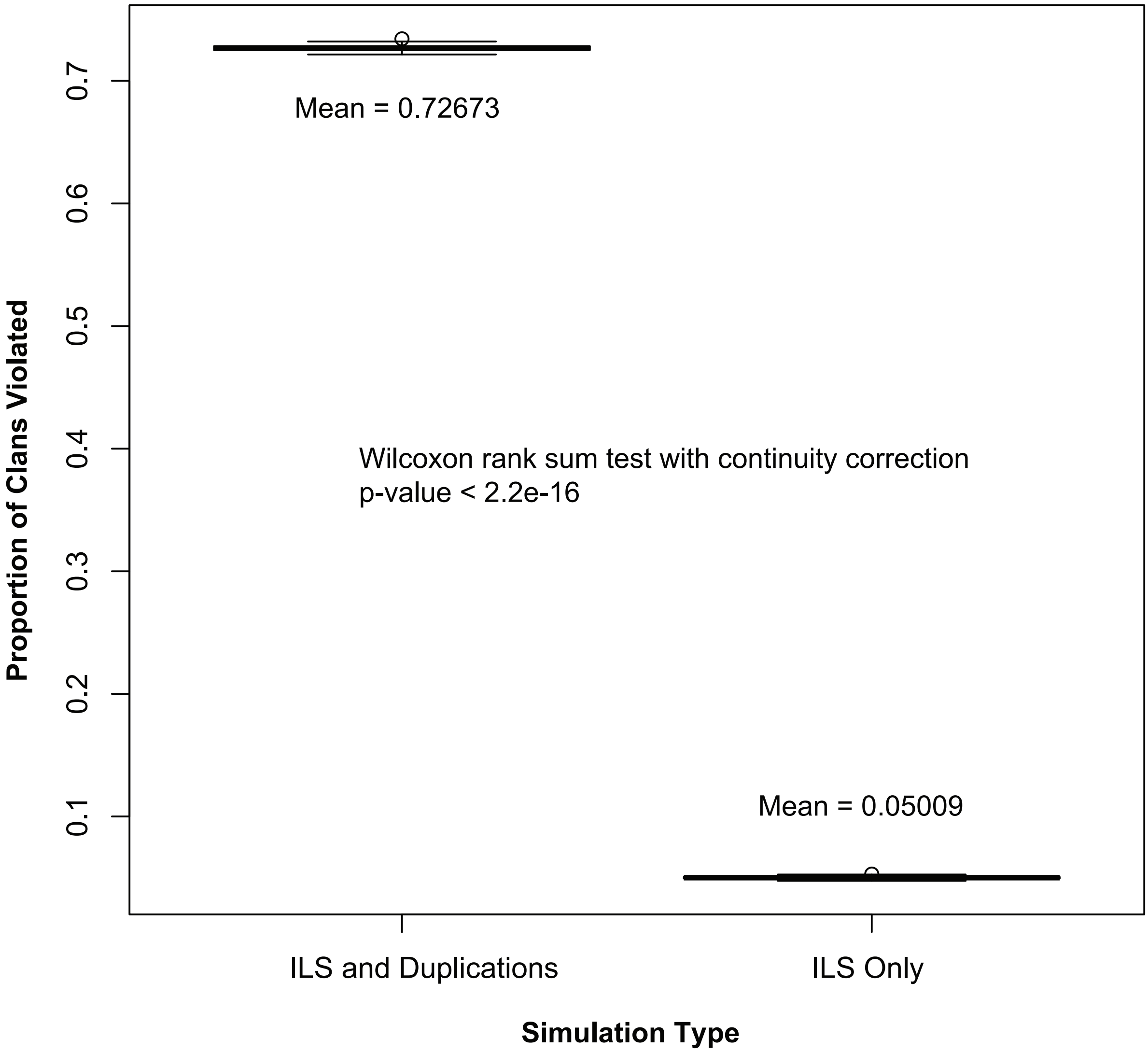

Supplementary Fig. S14. Boxplot of simulation results of Clan-Check in the presence of Incomplete Lineage Sorting (ILS) only or ILS with Early Duplications and Late Loss (100 reps).

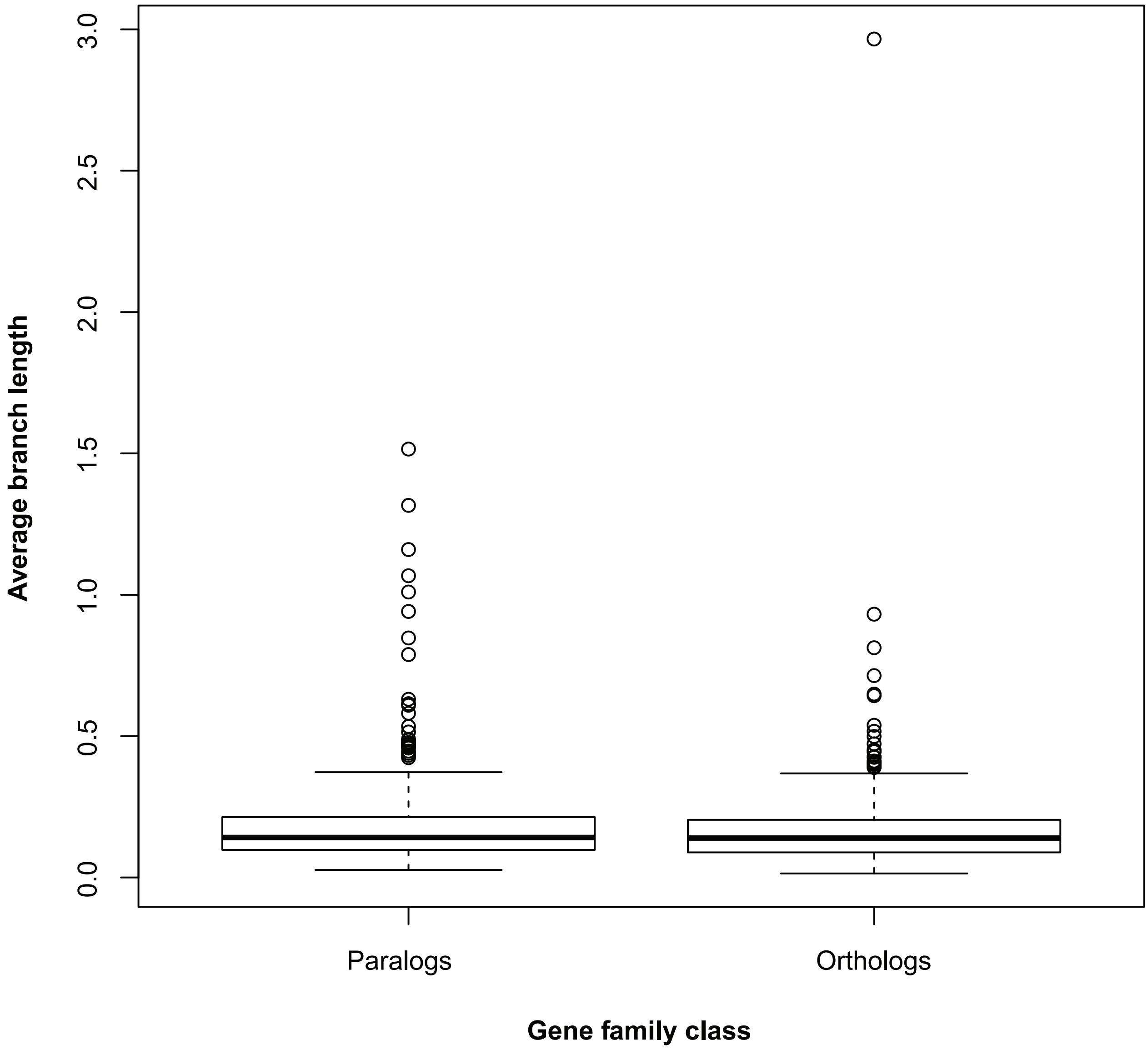

Supplementary Fig. S15. Average branch lengths of gene families classified as putative orthologs and paralogs by Clan-Check

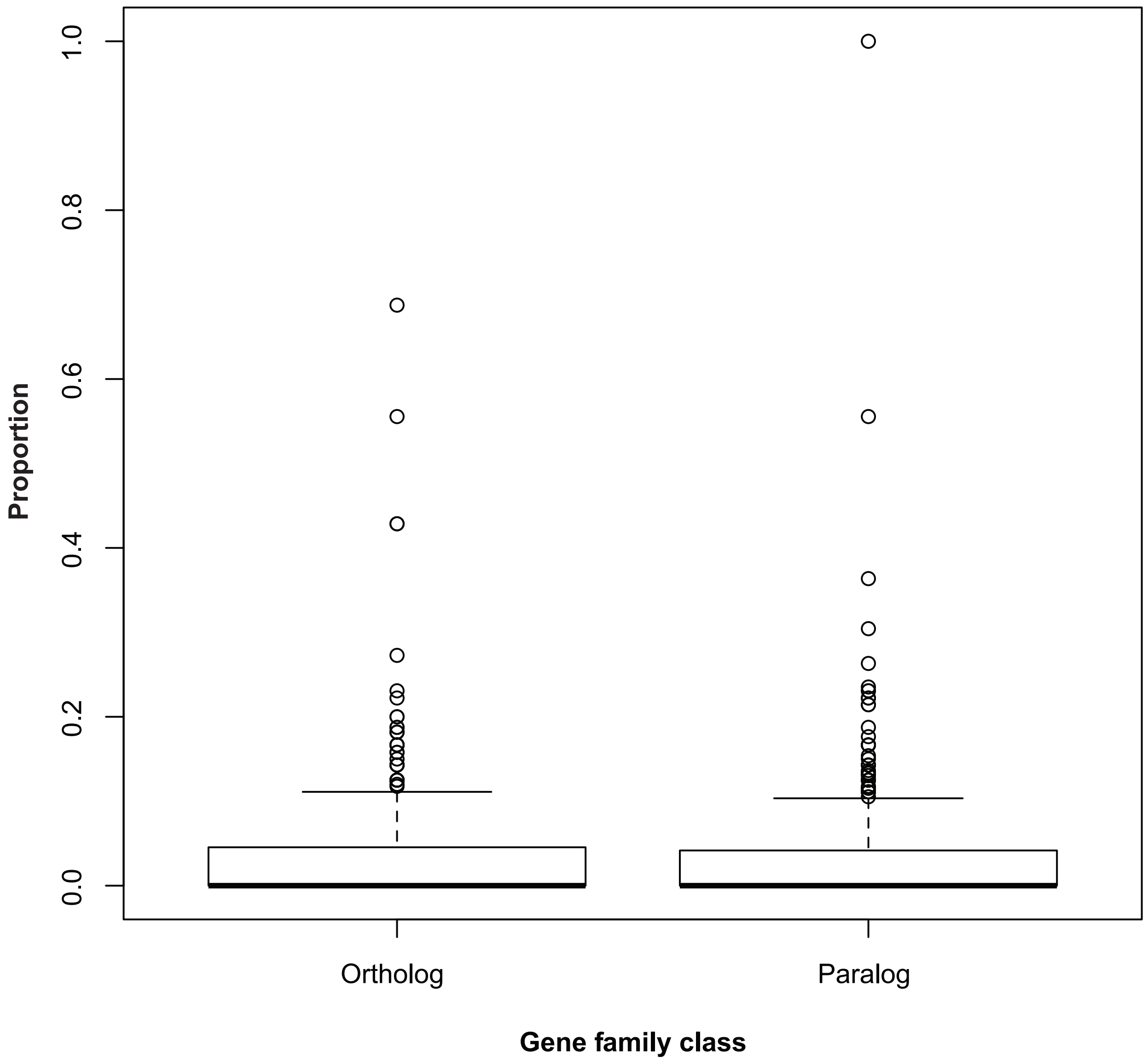

Supplementary Fig. S16. Comparison of the proportion of taxa that failed the compositional heterogeneity test after their classification as putative orthologs and paralogs from Clan-Check.

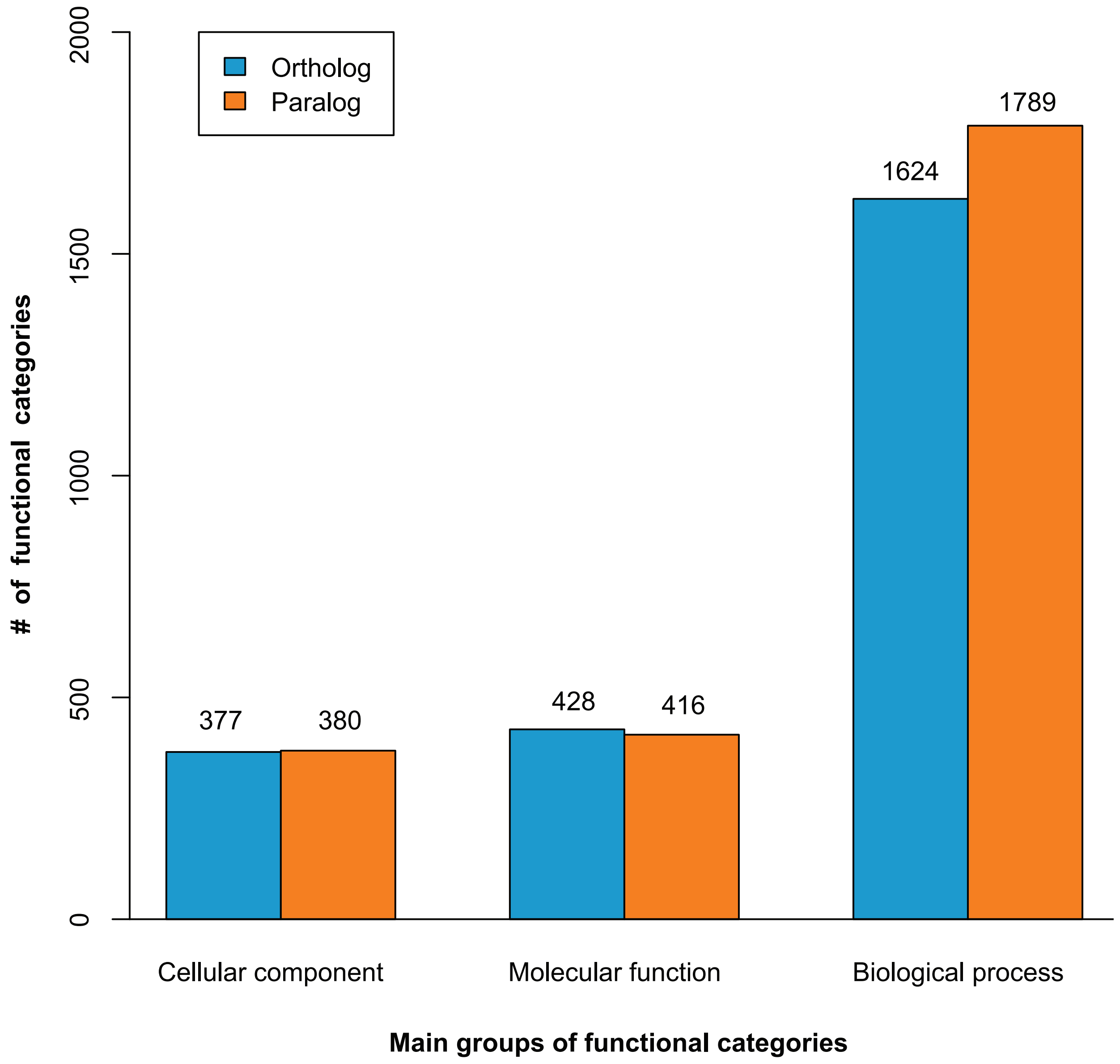

Supplementary Fig. S17. Comparison of main functional categories between putative orthologs and paralogs from Clan-Check.

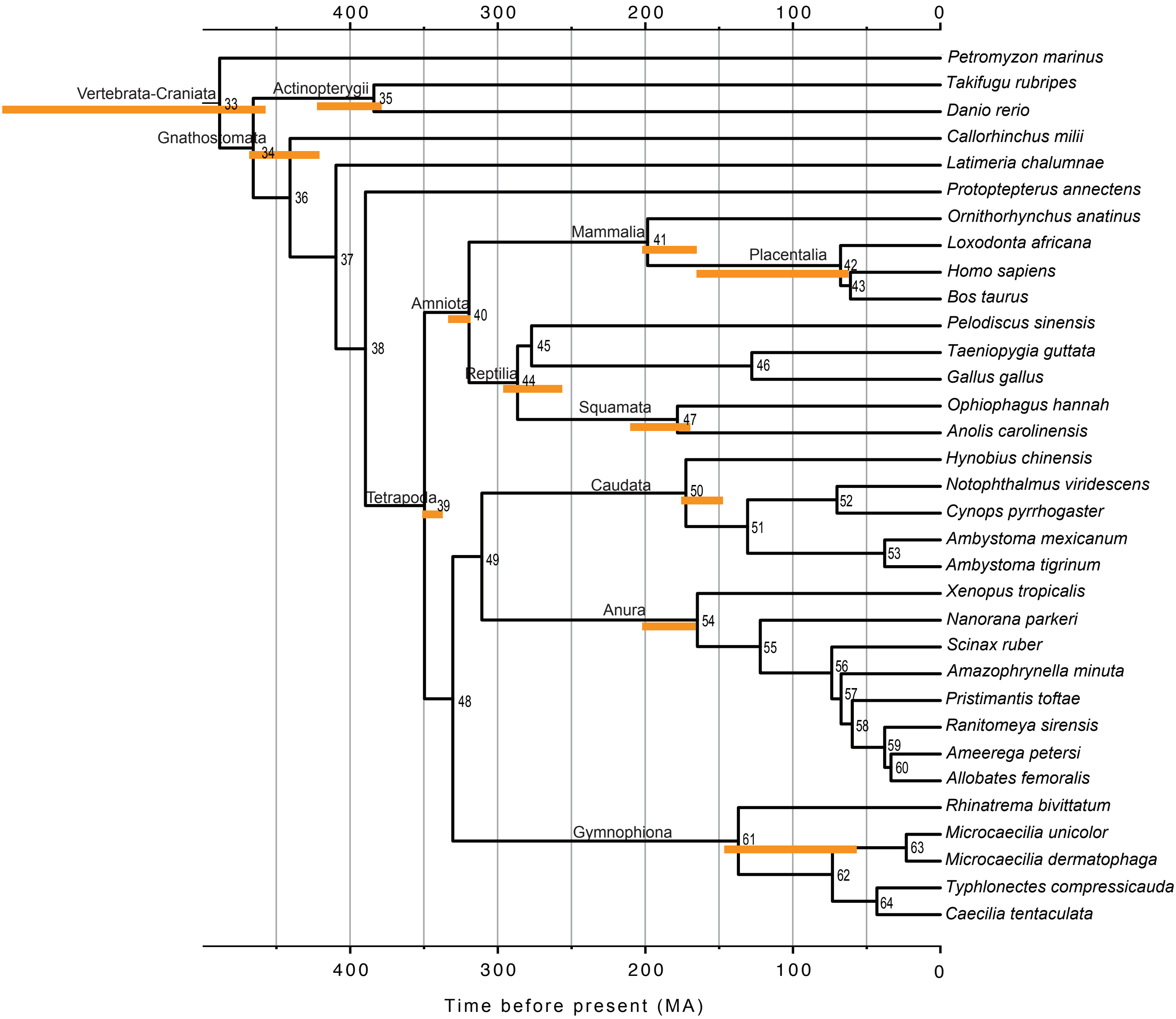

Supplementary Fig. S18. Timetree for Dataset 348 using Phylobayes. Node label numbers below branches correspond to Supplementary Table S3 and calibration ranges used (especified in Supplementary Table S6) are coloured in orange. Timescale in million years. See methods for more details.

Supplementary Table S3. Divergence times estimated for Dataset 348 using Phylobayes. Table of divergence time estimates with 95% Confidence Intervals for the nodes labelled in Supp. Fig. S18.

Node label	Mean Age	Standard Error	Inf95	Sup95
33	488.422	4.57545	481.086	496.928
34	465.624	3.06164	459.226	470.882
35	383.896	5.0695	376.402	395.294
36	440.707	4.55091	433.599	450.785
37	409.601	6.67461	397.867	419.013
38	389.606	4.16264	381.264	397.655
39	349.603	1.40998	346.148	351.638
40	319.407	1.46383	317.168	322.586
41	198.414	4.00119	188.583	204.24
42	67.6721	10.9624	52.8137	88.7575
43	60.9628	10.495	47.798	81.1955
44	286.545	2.96186	280.618	291.862
45	277.055	3.38867	269.937	283.062
46	127.827	14.8846	91.4406	145.073
47	178.207	7.0291	168.173	192.914
48	330.412	2.07376	325.819	334.067
49	310.728	2.82401	305.063	315.796
50	172.471	3.71821	162.954	177.022
51	130.664	6.7202	116.261	140.668
52	70.0814	10.3068	48.7022	87.3312
53	37.7724	9.8825	22.2936	56.9606
54	164.684	2.07433	159.965	168.691
55	122.06	3.267	115.413	128.987
56	73.6407	4.31858	66.3474	81.5789
57	67.2945	4.24311	60.4368	74.9928
58	59.7251	3.5404	53.7868	66.2106
59	37.7747	3.96415	32.0978	47.4017
60	33.4867	3.96949	26.8153	43.1129
61	136.893	7.71684	121.412	151.16
62	73.156	9.85178	57.3148	94.2496
63	23.1222	6.27723	12.9863	38.5273
64	43.0373	8.04296	28.7283	62.1037

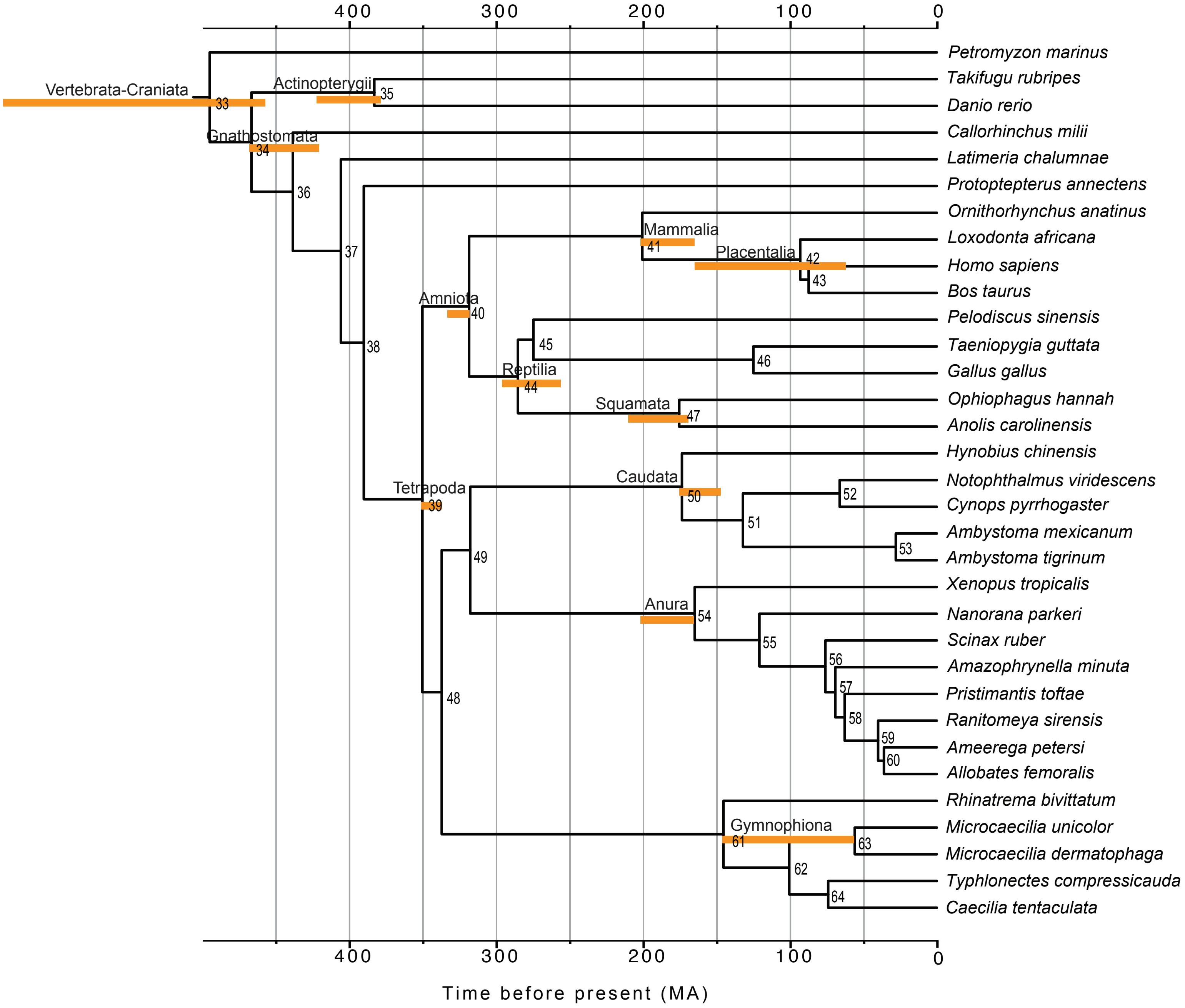

Supplementary Fig. S19. Timetree for Dataset 768 using Phylobayes. Node label numbers below branches correspond to Supplementary Table S4 and calibration ranges used (especified in Supplementary Table S6) are coloured in orange. Timescale in million years. See methods for more details.

Supplementary Table S4. Divergence times estimated for Dataset 768 using Phylobayes. Table of divergence time estimates with 95% Confidence Intervals for the nodes labelled in Supp. Fig. S19.

Node label	Mean Age	Standard Error	Inf95	Sup95
33	494.969	2.92462	489.378	501.138
34	466.591	2.47571	460.895	470.657
35	382.947	3.96739	375.311	390.253
36	438.373	2.84212	433.044	444.232
37	405.702	2.96252	399.902	411.786
38	390.086	3.14088	384.015	397.133
39	350.244	1.02198	348.064	351.966
40	318.393	1.09792	316.653	321.597
41	200.592	2.30553	194.542	204.99
42	93.0231	8.45044	80.5981	106.611
43	87.2498	7.86303	75.6122	100.269
44	285.176	4.45949	276.293	292.905
45	274.66	4.81497	266.232	283.411
46	124.897	12.2958	104.326	143.4
47	175.369	7.08783	167.502	192.076
48	337.03	1.20629	334.575	339.435
49	317.66	2.32494	312.345	321.3
50	173.54	2.67824	166.739	177.612
51	132.007	4.91611	123.547	141.051
52	66.1327	9.70155	47.6433	82.7467
53	27.9523	7.68796	17.2345	45.5489
54	164.802	1.99606	160.03	168.507
55	120.816	4.42399	111.284	128.394
56	75.8861	5.9407	64.8909	88.1292
57	69.126	5.11904	60.3623	80.0739
58	62.7335	4.50976	55.6934	72.5264
59	40.0079	3.2461	35.2735	47.4931
60	36.0511	3.11988	30.9476	43.2964
61	145.232	5.07125	135.432	156.321
62	100.555	7.05045	86.6792	114.306
63	55.8859	11.1968	33.4971	74.1374
64	73.99	9.47949	53.8639	90.3964

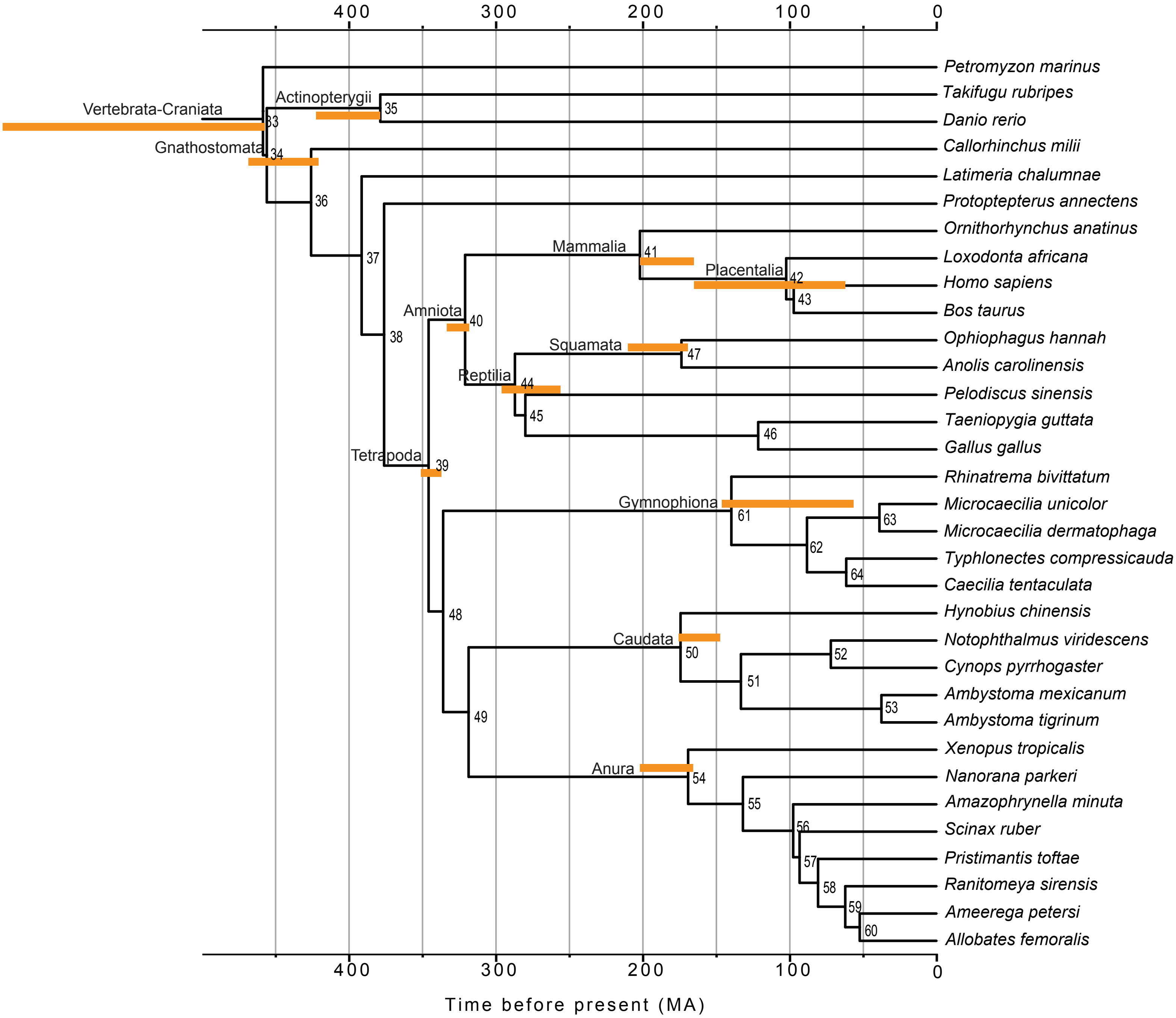

Supplementary Fig. S20. Timetree for Dataset 2656 using Phylobayes. Node label numbers below branches correspond to Supplementary Table S5 and calibration ranges used (especified in Supplementary Table S6) are coloured in orange. Timescale in million years. See methods for more details.

Supplementary Table S5. Divergence times estimated for Dataset 2556 using Phylobayes. Table of divergence time estimates with 95% Confidence Intervals for the nodes labelled in Supp. Fig. S20.

Node label	Mean Age	Standard Error	Inf95	Sup95
33	458.801	3.3881	453.324	464.475
34	456.196	3.29088	450.795	461.767
35	378.878	1.59156	375.723	382.363
36	426.014	4.94787	419.242	434.427
37	391.567	4.44593	384.499	399.113
38	376.282	3.68181	369.886	382.888
39	345.955	2.75733	341.539	350.844
40	321.089	2.12497	317.983	325.379
41	201.992	1.6471	199.306	205.999
42	102.316	1.19856	100.455	104.763
43	97.1788	1.23179	94.8413	99.5512
44	287.146	1.66443	283.126	290.212
45	280.049	1.8204	275.446	283.01
46	121.396	4.3102	111.222	128.034
47	173.682	4.22598	166.975	181.377
48	336.051	2.42221	332.04	340.53
49	318.71	1.68159	315.158	321.829
50	174.312	1.93886	171.218	179.218
51	133.172	3.7247	127.77	141.509
52	71.9191	4.79906	64.9214	82.4879
53	37.4378	5.58574	28.9833	49.4927
54	169.153	1.53535	166.113	172.552
55	131.817	1.61692	128.897	135.178
56	97.5599	1.35021	95.197	100.858
57	93.2051	1.46763	90.8623	96.5541
58	80.6233	1.54893	77.5165	83.5941
59	62.0801	2.45784	57.8647	66.0734
60	52.2203	3.08893	46.5728	57.5041
61	139.68	3.41711	133.291	145.548
62	88.1503	2.66056	83.2045	92.3102
63	38.8049	3.64651	32.7735	47.0941
64	61.4271	2.5559	57.0393	66.1163

Supplementary Table S6. Calibration time points (Mya) used in the timetree analyses.

Node	Node Maximum Age (Mya)	Node Minimum Age (Mya)	Source
Vertebrata-Craniata	636.1	457.5	(Benton et al. 2015; Ksepka et al. 2015)
Gnathostomata	468.4	420.7	(Benton et al. 2015; Ksepka et al. 2015)
Actinopterygii	422.4	378.19	(Benton et al. 2015; Ksepka et al. 2015)
Tetrapoda	351	337	(Benton et al. 2015; Ksepka et al. 2015)
Anura	201.5	165.3	(Benton et al. 2015; Ksepka et al. 2015)
Caudata	175.1	146.8	(Benton et al. 2015; Ksepka et al. 2015)
Gymnophiona	145.8	56	(Benton et al. 2015; Ksepka et al. 2015)
Mammalia	201.5	164.9	(Benton et al. 2015; Ksepka et al. 2015)
Placentalia	164.6	61.6	(Benton et al. 2015; Ksepka et al. 2015)
Squamata	209.5	168.9	(Benton et al. 2015; Ksepka et al. 2015)
Reptilia	295.9	255.9	(Benton et al. 2015; Ksepka et al. 2015)
Amniota	332.9	318	(Benton et al. 2015; Ksepka et al. 2015)

References

Benton MJ, Donoghue PCJ, Vinther J, Asher RJ, Friedman M, Near TJ. 2015. Constraints on the timescale of animal evolutionary history. Palaeontol. Electron.

Ksepka DT, Parham JF, Allman JF, Benton MJ, Carrano MT, Cranston KA, Donoghue PCJ, Head JJ, Hermsen EJ, Irmis RB, et al. 2015. The Fossil Calibration Database-A New Resource for Divergence Dating. Syst. Biol. 64:853–859.

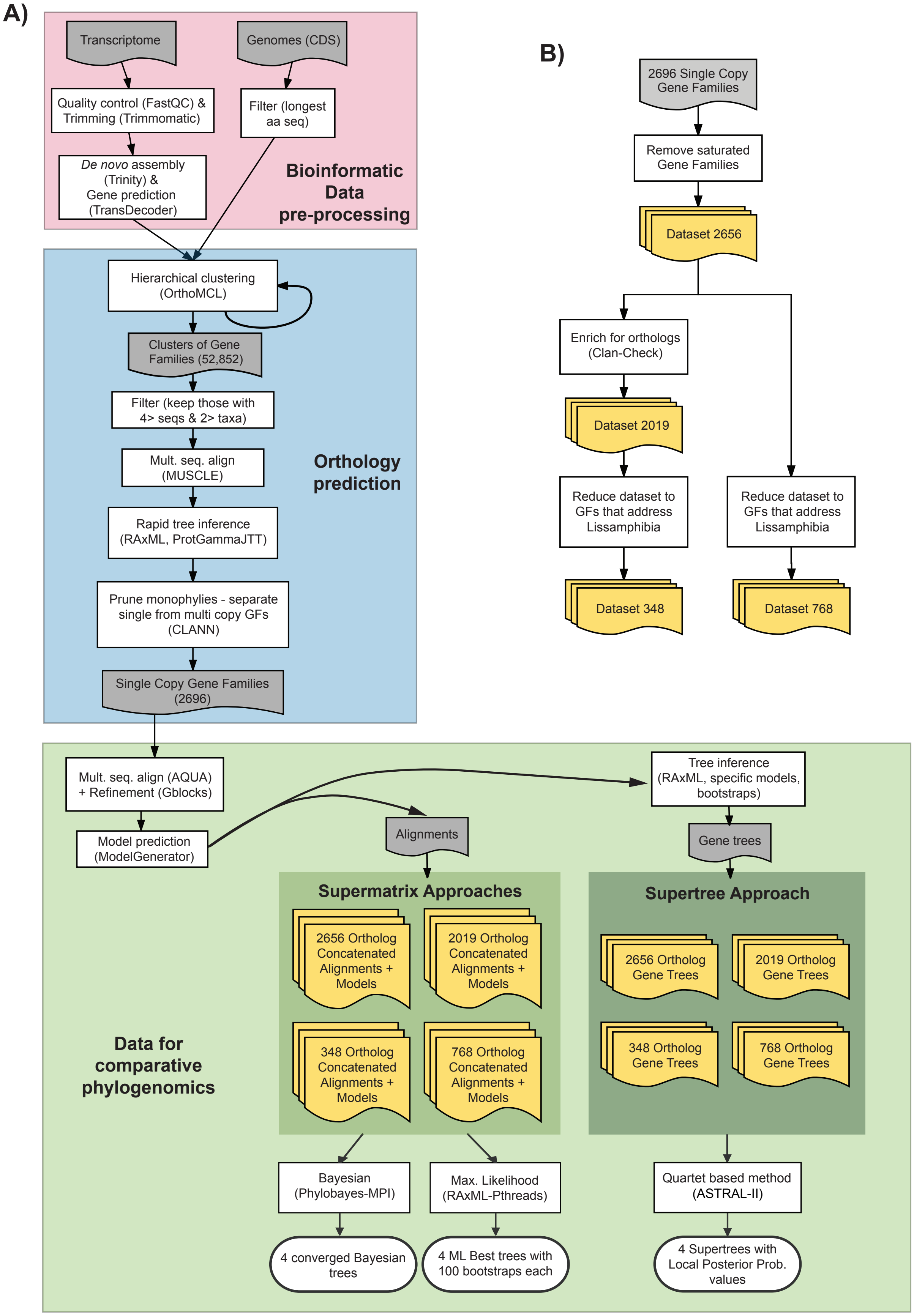

Supplementary Fig. S21. Schematic summary of data preparation for the phylogenomic study. A) Pipeline of bioinformatic processing carried out to infer orthologous gene families for the phylogenomic analyses for this study. B) Rationale of filters applied to produce Dataset 2656, Dataset 2019 and Dataset 348 analysed in this study, and an additional Dataset 768, set out to test the effect of no ortholog enrichment.
